# Prediction of Fitness to Drive in Patients with Alzheimer's Dementia

**DOI:** 10.1371/journal.pone.0149566

**Published:** 2016-02-24

**Authors:** Dafne Piersma, Anselm B. M. Fuermaier, Dick de Waard, Ragnhild J. Davidse, Jolieke de Groot, Michelle J. A. Doumen, Ruud A. Bredewoud, René Claesen, Afina W. Lemstra, Annemiek Vermeeren, Rudolf Ponds, Frans Verhey, Wiebo H. Brouwer, Oliver Tucha

**Affiliations:** 1 Department of Clinical and Developmental Neuropsychology, University of Groningen, Groningen, the Netherlands; 2 SWOV Institute for Road Safety Research, The Hague, the Netherlands; 3 CBR Dutch driving test organisation, Rijswijk, the Netherlands; 4 Department of Neurology, Alzheimer Center, VU University Medical Center, Amsterdam, the Netherlands; 5 Department of Neuropsychology & Psychopharmacology, Maastricht University, Maastricht, the Netherlands; 6 Department of Psychiatry and Neuropsychology, School of Mental Health and Neurosciences (MHeNS), Maastricht University, Maastricht, the Netherlands; 7 Department of Neurology, University Medical Center Groningen, Groningen, the Netherlands; Nathan Kline Institute and New York University School of Medicine, UNITED STATES

## Abstract

The number of patients with Alzheimer’s disease (AD) is increasing and so is the number of patients driving a car. To enable patients to retain their mobility while at the same time not endangering public safety, each patient should be assessed for fitness to drive. The aim of this study is to develop a method to assess fitness to drive in a clinical setting, using three types of assessments, i.e. clinical interviews, neuropsychological assessment and driving simulator rides. The goals are (1) to determine for each type of assessment which combination of measures is most predictive for on-road driving performance, (2) to compare the predictive value of clinical interviews, neuropsychological assessment and driving simulator evaluation and (3) to determine which combination of these assessments provides the best prediction of fitness to drive. Eighty-one patients with AD and 45 healthy individuals participated. All participated in a clinical interview, and were administered a neuropsychological test battery and a driving simulator ride (predictors). The criterion fitness to drive was determined in an on-road driving assessment by experts of the CBR Dutch driving test organisation according to their official protocol. The validity of the predictors to determine fitness to drive was explored by means of logistic regression analyses, discriminant function analyses, as well as receiver operating curve analyses. We found that all three types of assessments are predictive of on-road driving performance. Neuropsychological assessment had the highest classification accuracy followed by driving simulator rides and clinical interviews. However, combining all three types of assessments yielded the best prediction for fitness to drive in patients with AD with an overall accuracy of 92.7%, which makes this method highly valid for assessing fitness to drive in AD. This method may be used to advise patients with AD and their family members about fitness to drive.

## Introduction

Alzheimer’s disease (AD) is the most common aetiology of dementia and the number of patients with dementia is increasing rapidly [1,2]. AD is a progressive disease in which multiple cognitive domains are affected. In addition to the memory domain, also the domains of attention, visuospatial abilities, executive functioning, language and praxis are frequently impaired [3,4]. Impairments in these cognitive domains may influence many aspects of daily living, in particular the execution of complex tasks may be affected, such as driving a car [5].

Driving is a very meaningful instrumental activity of daily living and the preferred mode of transport of older adults [6]. Nevertheless, disabilities of old age could lead to an inability to drive a car safely. Driving cessation creates particular logistical problems for households of patients with AD [7]. While older drivers with other disabilities (e.g. cardiovascular diseases, muscular-skeletal conditions, visual impairment) may change to other modes of transport such as public transport by themselves, patients with AD typically need a responsible caregiver to travel with them [7], as many patients with AD experience a lack of orientation in public transport stations. Getting used to new transportation means (e.g. public transport) is cognitively more demanding in comparison to maintaining routine travel means (i.e. driving). Consequently, a large proportion of patients with AD depend on car driving to maintain independent mobility and autonomy [5,7]. There is a strong interest in maintaining mobility for patients with AD. However, safety risks for both the individual with AD as well as other road users have to be considered as well.

Previous research has shown that AD may impair driving [8–11]. A driver with AD might fail to recall road regulations and routes [12], may fail to oversee the infrastructure and perceive the distance to other vehicles, or may respond too slowly to the environment resulting in strategic and tactical errors, especially in non-automated situations [13]. Patients with AD are expected to become unable to drive safely in course of the disease and hence it is generally recommended that patients with severe AD (Clinical Dementia Rating (CDR) > 1) cease driving [14–17]. However, not all patients with AD are unsafe to drive [9–11]. Currently, AD is more frequently diagnosed in an earlier stage of the disease. Especially these patients with AD may be able to continue driving safely for several years after the diagnosis [14]. These findings indicate that it is necessary to investigate fitness to drive (FTDr) of patients with AD on a patient by patient basis.

On-road assessments are commonly used to investigate FTDr in many countries [18,19]. In an on-road assessment, an FTDr expert (e.g. from a driver’s licence authority) drives with a patient and judges whether the patient is driving safely. Considering the high and growing number of patients with AD, it becomes increasingly difficult to assess all patients with AD on the road soon after they have been diagnosed [20]. Other ways to evaluate FTDr are clinical interviews [21,22], neuropsychological assessments [14,23] and driving simulator rides [18,24].

Clinical interviews with both the patient with AD and a family member are regularly performed and certainly provide important information at clinical evaluation, since they may provide knowledge about previous accidents, near misses, fines, or changes in driving behaviour [21]. Nevertheless, one has to be cautious, because caregiver reports do not necessarily predict on-road driving performance [25]. AD has a relatively slow progression, therefore changes in driving performance may also be slow and difficult to detect for family members. In addition, family members who rely on the driver with AD for transportation may give biased reports [21].

Neuropsychological assessments are also frequently used for the evaluation of FTDr and include tests that assess cognitive functions known to be impaired in many patients with AD and that may affect driving. Performance on many neuropsychological tests has moderately high correlations with on-road performance, particularly tests of attention and visuospatial functioning [14,23]. Clinicians may use the results of these tests to help predict whether a patient with AD is driving safely [14], however, the accuracy of these predictions is often regarded as being too low [26,27]. Kay and colleagues (2012)[19] have suggested to aim for both sensitivity and specificity of at least 90%. Using a single test, such as clock drawing or the Trail Making Test, is probably not sufficient to reach this goal [28,29]. Combinations of tests may be more likely to predict FTDr than any single test [30], but even with multiple tests it is very difficult to achieve both high sensitivity and specificity for a binary classification of FTDr [26].

Driving simulators can mimic real-world driving in a controlled environment. Driving simulator rides can be seen as complex neuropsychological tasks. However, here the simulator outcome is categorized separately from neuropsychological assessments, because (1) the driving simulator used was not developed as a clinical tool to assess cognitive functions, but is an experimental tool for traffic research to measure driving behaviour, and (2) the technical and administrative set up of driving simulators and neuropsychological tests are different. Freund et al. (2002)[18] have shown that simulated driving correlates significantly with on-road driving in older adults, with and without cognitive impairments. Consequently, driving simulator rides may represent another method to predict on-road driving performance. However, it is not yet determined whether actual prospective accidents can be predicted with simulated driving [31]. Moreover, a large percentage of older drivers may not tolerate simulated driving due to motion sickness [32,33].

Using methods other than on-road assessments might have advantages. A routine clinical evaluation usually begins with a clinical interview using self- and informant reports. If cognitive impairments are reported during the interview, a neuropsychological assessment is frequently initiated. Consequently, clinical interviews and neuropsychological assessments represent cost-effective approaches which may be useful in the prediction of FTDr. Driving simulator rides are not part of standard clinical evaluations, but driving simulators are increasingly available in clinical units and research centres and driving simulator rides are safer and easier to conduct than on-road assessments. Currently, there is no standardised procedure to use these methods to evaluate FTDr. It is vital to determine the usefulness of these alternative methods in combination with one another.

The current study aims to develop a method to investigate FTDr in patients with AD in a clinical setting. The study includes three types of assessments for the prediction of on-road driving performance, i.e. clinical interviews, neuropsychological assessments and driving simulator rides. In addition, all participants were evaluated using an on-road assessment (criterion). The goals of the study are threefold. The first goal is to determine for each of the three types of assessments separately which combination of measures are most predictive for on-road driving performance. Second, the predictive value of the clinical interviews, neuropsychological assessment and driving simulator rides are compared with one another to determine which type of assessment is most useful for the prediction of FTDr. Third, the predictive accuracy of FTDr is determined when using the best possible combination of clinical interviews, neuropsychological assessment and/or driving simulator rides.

## Materials and Methods

### Participants

#### Patients with Alzheimer’s disease

Participants with AD (n = 81) were assessed at five locations in the Netherlands; at two hospitals, two nursing homes and a university, in 2013 and 2014. Inclusion criteria for patients were an age above 30, a diagnosis of AD and a wish to continue driving. AD was diagnosed by a neurologist, geriatrician, psychiatrist or general practitioner. All participants held a current valid driver’s licence. Exclusion criteria were the diagnosis of other neurological or psychiatric conditions that may influence driving performance and usage of medications with a severe influence on driving ability.

Of the 81 patients with AD, 71 participants (87.7%) met criteria for probable AD and 10 participants (12.3%) met criteria for both probable AD and vascular dementia (mixed dementia) [34,35]. Patients were aged 52 to 91 years (mean = 72.3 years; SD = 9.4 years) and 53 (65.4%) of the patients were men. Patients had held a driver’s licence for 25 to 73 years (mean = 49.8; SD = 9.5 years) and the estimation of their total distance driven ranges from 107,000 to 15,230,000 km (mean = 1,426,000; SD = 2,867,000 km).

#### Healthy participants

Furthermore, 45 healthy individuals participated in the study. Inclusion criteria for healthy participants were an age above 70, no diagnoses of psychiatric or neurological conditions, no diagnoses that would require referral to the Dutch driving test organisation, no usage of medications with a severe influence on driving ability and a wish to continue driving. The age limit for healthy participants was higher than for patients to avoid having a healthy sample that is younger than the patient sample. All healthy participants also held a current valid driver’s licence. Healthy participants were aged 70 to 87 years (mean = 76.3; SD = 4.7 years) and 24 (53.3%) healthy participants were men. Healthy participants had held a driver’s licence for 7 to 63 years (mean = 51.1; SD = 8.6 years) and the estimation of their total distance driven ranges from 22,000 to 7,213,000 km (mean = 1,258,000; SD = 1,435,000 km).

Table 1 presents characteristics of patients with AD and healthy participants. As expected, a higher proportion of patients with AD had a CDR-score of 0.5 or 1 compared to healthy participants (χ^2^ = 112.5; df = 2; p < .001). Correspondingly, patients with AD had a lower score on the Mini Mental State Examination (MMSE) (U = 131.0; p < .001; r = 0.771) than healthy participants. Other characteristics did not differ significantly between patients with AD and healthy participants.

**Table 1:** Characteristics of healthy participants and patients with Alzheimer’s disease.

	Group		
Characteristics	Healthy (n = 45)	AD (n = 81)	*P* Value (df)
Age, mean (SD), y	76.3 (4.7)	72.3 (9.4)	.105 ^a^ (125)
Male sex, No. (%)	24 (53.3%)	53 (65.4%)	.189 ^b^ (1)
Education, mean of 7 stages (SD)	5.2 (1.3)	4.9 (1.4)	.129 ^a^ (6)
CDR-score, No. (%)			**< .001** ^c^ (2)
0	42 (93.3%)	1 (1.2%)	
0.5	3 (6.7%)	67 (82.7%)	
1	0 (0.0%)	13 (16.1%)	
MMSE-score, mean (SD)	28.8 (1.1)	23.2 (3.7)	**< .001** ^a^ (125)
Cholinergic medication, No. (%)	NA	36 (44.4%)	
Cholinergic medication dose, mean (SD), mg/day	NA	12.7 (5.7)	
Other medication affecting the CNS, No. (%)	3 (6.7%)	8 (9.9%)	1.000 ^b^ (1)
Driving experience, mean (SD), y	51.1 (8.6)	49.8 (9.5) ^d^	.378 ^a^ (122)
Driving experience, mean (SD), km	1,258,000 (1,435,000)	1,426,000 ^d^ (2,867,000)	.201 ^a^ (122)
Car accident in past year, No. (%)	3 (6.7%)	5 (6.2%)	1.000 ^b^ (1)
Traffic fine in past year, No. (%)	9 (20.0%)	17 (21.0%)	.882 ^c^ (4)

^a^ Mann-Whitney U test

^b^ Fisher’s Exact test

^c^ χ^2^ test

^d^ For 78 patients out of 81 patients, because 3 patients did not report the information.

Abbreviations: AD, Alzheimer’s disease; Education, Verhage scale for the Dutch educational level ranging from 1 (primary school not finished) to 7 (university level); CDR-score, Clinical Dementia Rating Total Score; MMSE-score, Mini Mental State Examination Total Score; NA, not applicable; CNS, central nervous system; Other medication affecting the CNS include benzodiazepines, antiepileptic drugs, antidepressants and pain killers.

### Measures

The subsequent description of the methods comprises only tests and measures which were considered in the present study. The preselection of measures was based on the literature and intended to cover relevant cognitive domains (e.g. attention, executive functioning and visuospatial functions), with no redundancy [21,36–39]. For a full description of the study protocol, please see S1 Appendix.

#### Clinical interviews and ratings

Clinical interviews and ratings consisted of the CDR and a driving questionnaire, and involved both the participant and an informant (e.g. the participant’s partner).

Participants were requested to complete a *driving questionnaire* (adapted from the Safe Driving Behaviour Measure [37]). The questionnaire consists of three parts: a demographical profile (7 items), a driving profile (23 items) and safe driving behaviour queries (54 items). For this study, one item of the driving profile was used, i.e. the kilometres driven in the previous twelve months representing recent driving experience. The question was categorical with the following answer options: *less than 1*.*000 km* (1), *1*.*000–5*.*000 km* (2), *5*.*000–10*.*000 km* (3), *10*.*000–20*.*000 km* (4), *20*.*000–30*.*000 km* (5), *30*.*000–50*.*000* (6), *more than 50*.*000 km* (7). In addition, a total score for safe driving behaviour was calculated. Each safe driving behaviour item was a driving situation that could be rated on a five-point scale ranging from *not difficult* (0) to *impossible to do* (4), or as *not applicable* (no score). A mean score was calculated by summing up all scores divided by the number of items endorsed. In addition to the driving questionnaire, both the informant and the participant were asked whether the participant is *still driving as safely as when the participant was middle-aged* (1), is *driving less safely compared to when the participant was middle-aged* (2) or *drives unsafely* (3). They were also both asked whether they believed that the participant should cease driving, given the response alternatives: *no* (1), *questionable* (2) or *yes* (3).

The *CDR* [40] consists of six subscales: *memory*, *orientation*, *judgement & problem solving*, *community affairs*, *home & hobbies* and *personal care*. Items of all six subscales are discussed with the informant. Subscales *memory*, *orientation* and *judgement & problem solving* also contain items to discuss with the participant. For each subscale, a subscore was determined: 0 (no impairment), 0.5 (questionable impairment), 1 (mild impairment), 2 (moderate impairment) or 3 (severe impairment). The CDR total score was calculated with the Washington University’s CDR-assignment algorithm [40] giving a total score of 0, 0.5, 1, 2 or 3. Moreover, the CDR sum of boxes score was calculated by summing up the six subscores.

#### Neuropsychological assessment

A neuropsychological test battery was composed aiming to measure cognitive functions that are known to be important for driving, containing aspects of attention, executive functioning and visuospatial abilities [21,36–39,41–43]. The neuropsychological tests included both paper and pencil tests as well as computerized tests.

The *MMSE* [44,45] was used as a general measure of cognition. The MMSE assesses basic abilities of a range of cognitive functions including memory, attention and language skills. The MMSE is widely used as a screening tool for dementia [46]. The sum score ranging from 0 to 30 was calculated.

The *Trailmaking Test (TMT) A* and *B* [47] was performed as a measure of cognitive flexibility. The TMT consists of two parts, TMT A and TMT B. In TMT A, participants are instructed to draw lines between numbers presented on a paper in ascending order as fast as possible. An upper limit was set at five minutes. The time to completion was measured. In TMT B, participants have to draw a line between numbers and letters in ascending order, alternating between both types of stimuli as fast as possible. An upper limit was set at six minutes. The time to completion was measured. In an attempt to remove the effects of simple sequencing, visual scanning and psychomotor functioning, the time to complete TMT A was subtracted from the time to complete TMT B (TMT B-TMT A) and this index score was taken as a measure of cognitive flexibility [48].

*Drawings* [39] were included as a measure of visuoconstructive ability. Participants were asked to draw from memory a house, a star with five points, a cube and a clock on paper. For each drawing a maximum of two points was scored if the object was recognizable and complete, resulting in a total score between 0 and 8.

Two *Mazes*, suggested as predictors of high crash risk in older drivers by Staplin and colleagues (2013)[38], were included as a measure of visual orientation. The mazes were provided on paper. One practice maze of intermediate difficulty was completed before the two test mazes were administered. Maze 1 was labelled by the author as “easy”, whereas maze 2 was labelled as “difficult” [38]. For the administration of each maze, the experimenter pointed at the starting point of the maze and instructed the participant to find the exit of the maze by drawing a continuous line from the starting point to the exit. In case of errors, participants were instructed to follow the line they incorrectly drew backwards until they could continue the correct route. The time to complete each maze was measured.

The *Adaptive Tachistoscopic Traffic Perception Test (ATAVT)* of the Vienna Test System (VTS)[49] was used to assess the ability to gain an overview in traffic situations. Photographs of traffic situations were shown to the participants for approximately one second per picture on a computer. Afterwards, the participants were asked to report what was in it, choosing at least one out of five answer options: pedestrians, cars, (motor)cyclists, traffic signs and traffic lights. Photographs were presented adaptively, meaning that after an initial phase, the difficulty of the items was increasingly tailored to match the ability of the participant. The outcome measure was a performance parameter based on the 1PL Rasch model, provided by the VTS.

A *traffic theory test* was developed by the SWOV Institute for Road Safety Research and the CBR Dutch driving test organisation to measure knowledge about traffic theory. A total of 28 pictures presenting traffic scenes were consecutively displayed on a computer screen. For each scene, participants were requested to answer a question regarding the meaning of traffic signs, priority regulations and other traffic rules. There was a time limit of twelve seconds per question. The number of correct answers and the mean response time were registered.

A *hazard perception test* was used to measure hazard perception ability [50]. Traffic situations were presented by a computer as photographs taken from the driver’s point of view. The current driving speed was also shown. Participants had to decide whether they would brake, release the gas pedal or maintain their speed in 25 traffic situations. There was a time limit of eight seconds for each traffic situation. This test requires timely planning and decision making in an applied context of driving situations [50]. The number of correct answers and the mean response time were measured.

*Reaction Time (RT) S1* and *S2* (VTS, Schuhfried) [51] are computer tests that measure visual and auditory attention respectively. In RT S1, participants have to look at a black circle and when the circle turns yellow they have to respond as quickly as possible by pressing a button. In RT S2, participants have to respond as quickly as possible to a tone at 2000 Hz by pressing a button. In both tests, participants have to keep their index finger on a rest button until a stimulus is presented, then they should lift their finger from the rest button to press the reaction button. The reaction time (RT) is the time between the appearance of the stimulus and the moment the finger leaves the rest button. Motor time (MT) is the time that elapses between the moment the finger leaves the rest button and the moment that the reaction button is pressed. Mean RT, mean MT, as well as the standard deviations of RT and MT were measured.

*RT S3* (VTS, Schuhfried) [51] was included as a measure of inhibition. In the RT S3, a sequence of yellow and red lights, a tone and combinations of these stimuli was presented. The critical combination to which the participant was instructed to respond was the stimuli from the RT S1 (yellow circle) and S2 (tone). When both, a yellow circle and a tone, were presented, participants had to press the reaction button as quickly as possible. If only one of the stimuli was presented or a red circle was shown, participants had to inhibit their responses. Similar to RT S1 and S2, the mean RT, mean MT as well as the variability of RT and MT were measured.

#### Driving simulator rides

Five fixed-base Jentig50 driving simulators of ST Software were used at five locations in the Netherlands. The simulators consisted of an open cabin mock-up with a steering wheel, gear box, gas pedal, brake pedal, clutch and simulated driving sound. Three 50 inch LED screens provided the participant with a view on the road, a view of 200° in total. The dashboard, car windows, side mirrors and rear view mirror were realized on the screens. During driving the participants wore the safety belt. Graphical rendering, traffic simulation and system control showing a user interface for the simulator operator were running on computers. The graphical interface was designed with StRoadDesign (ST Software) and the scenario was programmed with scripting language StScenario (ST Software). Simulated traffic was able to adapt to the behaviour of the participant [52].

Different driving simulator rides were used to assess various aspects of driving behaviour that are assumed to be important for safe driving and that could be predictive for FTDr. After a short practice ride, four test rides were employed, i.e. the *Lane tracking ride*, *Intersections a*, *Intersections b*, and the *Merging ride*. All rides were driven with automatic transmission. Participants were instructed to behave as they would drive a real car. There was a practice ride to get acquainted with the driving simulator, especially with steering. This ride was in a rural environment on a slightly winding road with oncoming traffic on the left lane. The speed was regulated by the computer and increased stepwise up to 100 km/h. The first test ride (Lane tracking ride) was in the same rural environment, but the participants were in control of their own speed. There was no speed limit. In the Lane tracking ride, participants were asked to choose a comfortable speed, after which they were requested to drive as if they were in a hurry. The average speed as well as swerving, as indicated by the standard deviation of the lateral position (SDLP), was measured twice, i.e. when participants were driving at a comfortable speed (*Speed of choice*, *SDLP*) and when they were in a hurry (*Speed in hurry*, *SDLP in hurry*). Additionally, the number of collisions (*Number of collisions*) was registered during the Lane tracking ride. The second and third test ride (Intersections a and b) were identical to each other. This ride was repeated, because it was taken into consideration that participants may need help in identifying traffic signs and intersections in the driving simulator at the beginning. The intersections ride was in a rural environment, but now the participant encountered intersections with different priority regulations. In both Intersections a and b, three intersections were analysed where the participant had to give way, including one with traffic lights. The participant was always driving straight ahead. There was oncoming traffic on the left lane, but also traffic coming from left and right at intersections. Furthermore, at a certain point, a car suddenly pulls out of a parking lot in front of the participant. Speed limits differed between 60 and 80 km/h. The participants were asked to obey the traffic rules. In the first intersections ride (Intersections a) and in the repetition of the intersections ride (Intersections b), measures were (1) lowest speed when approaching three intersections where the participant has to give way (*Minimum speed Int 1*, *2*, *3*), (2) average deviation from the speed limits (*Dev from speed limit*), (3) brake reaction time when the traffic lights turn yellow (*RT traffic lights*), (4) whether or not the participant brakes for the car that pulls out of a parking lot (*Braking for car that pulls out*) and (5) the total number of collisions (*Number of collisions*) in the respective intersections ride. In the fourth and final test ride (Merging ride), the participant merged into a crowded motorway with two lanes in each direction and was asked to overtake one vehicle and subsequently leave the motorway. Measures were (1) speed while merging (*Speed while merging*), (2) deceleration of the rear car right after merging (*Deceleration rear car*), (3) time headway to the car in front right after merging (*Time headway merging*) and (4) the smallest time headway to any car in front during the Merging ride (*Minimum time headway*)[53].

Participants were instructed to report to the researchers if they were not feeling well during driving. After each ride, a researcher asked how the participant was feeling. If symptoms of simulator sickness, such as dizziness or nausea, were reported or observed, participants were advised to take a break and if their symptoms did not disappear to abort the driving simulation.

#### On-road driving assessment

The on-road driving assessment was carried out in the participant’s own car during daylight hours. The on-road driving assessments were rated by approved experts on practical FTDr of CBR, the Dutch driving test organisation, experienced in the assessment of people with impairments like dementia. The experts are extensively trained to evaluate the effects of impairments on driving behaviour. They were blind to the participant’s diagnosis, clinical ratings, neuropsychological test results, as well as driving simulator performance. However, they did know that the participant could have cognitive problems because they were using a specific protocol for cognitive impairment. They made use of the Test Ride Investigating Practical fitness to drive (TRIP) forms [11,54]. The TRIP consists of 60 items, concerning lateral positioning, gap distances, speed, visual behaviour, responses to traffic signs, overtaking, anticipation, communication, turning left, merging, technical execution and perception and insight, each rated as either *sufficient*, *doubtful*, or *insufficient*. Finally, one overall score was given by the expert on practical FTDr, resulting in a *pass*, *doubtful* or *fail* outcome. This variable was recoded into a dichotomous item which indicates whether or not a participant is fit to drive (*FITtoDRIVE*): pass outcomes indicated fitness to drive while doubtful or fail outcomes indicated that participants are unfit to drive.

### Procedure

Participants were invited to take part in the study on a voluntary basis. Patients were recruited via multiple health care centres and from the general community by means of advertisements. Healthy participants were recruited from the general community by means of advertisements and word of mouth. The study was conducted according to ethical guidelines and was approved by the Medical Ethical Committee at the University Medical Center Groningen (METc 2012/172, ABR-nr. NL39622.04212) and the Ethical Committee Psychology at the University of Groningen (ppo-013-045 & ppo-012-065), the Netherlands. All participants provided their written informed consent to participate in the study. Patients were regarded able to consent themselves, since all patients were in a mild stage of dementia. In addition, verbal consent was asked from the participant and the informant when the study was explained verbally at the start. The Ethical Committees approved this consent procedure. Patients received no direct reward for participation, but patients who passed the on-road driving assessment could use this outcome in an official relicensing procedure. Healthy participants were rewarded 15 Euros for participation.

Participants were invited twice. On the first occasion, clinical interviews with the participant and an informant were conducted, as well as the neuropsychological assessment and the driving simulator rides. On the second occasion, the on-road driving assessment took place. The participant invited an informant of their choice, which was in most cases their partner and otherwise one of their children, caretakers or friends. Participants were instructed to fill out the driving questionnaire with the help of the informant beforehand and bring the questionnaire with them to the first session. During that first session, an interviewer conducted the clinical interview with the informant in absence of the participant. The driving questionnaire was discussed with the informant in case of ambiguous items. Meanwhile an experimenter instructed the participant for the neuropsychological assessment. After the neuropsychological assessment, the interviewer conducted the clinical interview with the participant in absence of the informant. Finally, the driving simulator rides were performed.

During the first session, participants were also screened to assure that they met the minimum requirements for the on-road driving assessment with regard to visual and motor functions. Reported medication use was also checked for not using category 3 medications, which are classified as having a severe influence on driving ability [55]. The first session lasted approximately four hours in total, including around half an hour driving simulation. The second session, the on-road driving assessment, was performed by the participant on another day and took around 45 minutes.

### Statistical analyses

#### Data cleaning and missing value analysis

Analyses were performed with IBM SPSS Statistics 22. The few missing values of the *clinical interviews* were not replaced. Missing values occurred in the judgement of the informant about how safe the participant drives and the opinion of the informant whether the participant should cease driving (two cases) and the mean score for safe driving behaviour (six cases).

Missing values occurred in the *neuropsychological assessment* when participants exceeded a certain number of incorrect responses or failed to complete a test within the given time limits. These missing values were imputed by the worst scores of the respective group that did complete the test. Such imputations were made for TMT A (one case), TMT B (31 cases), Maze 1 (two cases) and RT S3 (three cases).

Twenty-three participants (28.4%) with AD reported feelings of dizziness or nausea indicating simulator sickness and were excluded entirely from analyses that involved *driving simulator rides*. Driving simulator data of two other participants with AD were missing; one patient was unable to steer the driving simulator, the other patient was panicking in the driving simulator and both patients had to stop driving. Due to a technical error, driving simulator data of the third ride of one patient are missing. Twenty-four healthy participants (53.3%) reported simulator sickness and were excluded entirely from analyses that involved driving simulator rides. Four driving simulator variables (see explanation below) were occasionally missing although the participant had driven all driving simulator rides. In the group of patients, in both intersection rides, the brake reaction time to the traffic lights was missing in 11 cases. In the healthy comparison group, this variable was missing in five cases in the first intersection ride only. These missing values are omission errors of participants who did not notice the traffic lights, therefore the worst scores of the respective group that did brake were inserted. If participants merged on the motorway after all cars had passed, there are no values for the deceleration of the rear car. In the group of patients, these missing values occurred in seven cases and values were inserted using an imputation model (including all complete variables of the driving simulator) that was estimated by maximum likelihood (ML), providing a singly imputed dataset.

#### Group comparisons

Patients with AD and healthy participants were compared using separate MANOVAs for the clinical interviews, neuropsychological assessment and driving simulator rides. Significance level alpha was initially set to .05. However, alpha level was adjusted by using Bonferroni-Holm correction in order to control for alpha error inflation in multiple comparisons. Furthermore, effect sizes were calculated for multivariate and univariate comparisons (indicated by η^2^ and Cohen’s d). The index η^2^ provides information about the proportion of variance which is accounted for by the multivariate comparisons. As described by Cohen [56], η^2^ is a function of the effect size index f. According to Cohen [56], a small effect size (f = .10) corresponds to an η^2^ = .0099, a medium effect size (f = .25) to an η^2^ = .0588 and a large effect size (f = .40) to an η^2^ = .1379. The effect size Cohen’s d was computed for univariate comparisons. Negligible effects (d < 0.20), small effects (0.20 < d < 0.50), medium effects (0.50 < d < 0.80) and large effects (d > 0.80) were distinguished. A similar procedure was performed to compare patients who passed the on-road driving assessment with patients who failed the on-road driving assessment.

#### Development of prediction models for fitness to drive

The goal of the present analysis was to predict fitness to drive as indicated by the dichotomous variable FITtoDRIVE. Prediction is a statistical term meaning that the dependent variable FITtoDRIVE is hypothesized to be influenced by the independent variables. The independent variables (predictor variables) were divided into three groups, e.g. clinical interviews, neuropsychological assessment and driving simulator rides, in order to explore the accuracy of each group of variables in predicting fitness to drive. For each group of predictor variables, the first analyses involved correlations with FITtoDRIVE (point biserial correlation coefficients). Predictor variables correlating significantly (p<0.05) with FITtoDRIVE were selected for the second analyses, i.e. binary logistic regressions with a forward inclusion method. Binary logistics were performed to determine the validity of each respective group of predictor variables in predicting FITtoDRIVE. In order not to lose predictive power, a liberal entry criterion for predictor variables (p<0.20) was used. Predictor variables included by the regression model were used in the third step, i.e. Discriminant Function Analysis (DFA). In DFA, selected predictor variables are weighted determining their utility to distinguish between fit and unfit to drive (FITtoDRIVE). A prediction equation for each group of predictors was developed by summing up the predictor variables multiplied with the corresponding unstandardized discriminant coefficients. This resulted in three predictor variables representing the clinical interviews, the neuropsychological assessment and the driving simulator rides. Finally, receiver operating characteristic (ROC) analyses were carried out to test the accuracy of the developed variables in predicting FITtoDRIVE. In an ROC analysis, a curve is created by plotting the sensitivity against the specificity of the predictor variable. The area under the curve (AUC) is used as a classification measure with larger areas indicating better predictive accuracy.

#### Comparison and combination of prediction models

In order to compare the three prediction models described above, patients with data of the complete assessment procedure, including clinical interviews, neuropsychological assessment and driving simulator rides, were selected (n = 55). On this group of patients, three separate ROC analyses were performed using the three developed predictor variables explained above. The resulting AUCs give information about which predictor variable holds the best predictive accuracy. Furthermore, the three predictor variables were entered in a hierarchical binary logistic regression analysis to investigate whether using more than one type of assessment would improve the predictive accuracy. According to the common procedure in clinical practice, the predictor variable of clinical interviews was entered in block 1, the predictor variable of neuropsychological assessment was entered in block 2 and the predictor variable of driving simulator rides was entered in block 3. If neuropsychological test performance or driving simulator performance were shown to significantly add predictive validity in hierarchical logistic regression, DFA was performed as described above to create a final predictor variable for the prediction of FITtoDRIVE integrating all information from the three types of assessments. This final predictor variable was used in an ROC analysis which revealed the accuracy of classification of the complete method. The predictive accuracy was further evaluated by calculating the sensitivity, specificity, positive predictive power and negative predictive power using various cut-offs. In order to create one number showing the highest combination of sensitivity and specificity, Youden’s indexes were calculated by sensitivity + specificity -1 [57].

## Results

### Group comparisons between healthy individuals and patients with AD

Significant differences were found between patients with AD and healthy participants with regard to clinical interviews (Wilk’s lambda = 0.401, F(12,103) = 12.841, p < .001, η^2^ = .599), neuropsychological assessment (Wilk’s lambda = 0.347, F(23,97) = 7.926, p < .001, η^2^ = .653), and driving simulator rides (Wilk’s lambda = 0.488, F(22,55) = 2.624, p = .002, η^2^ = .512). In the clinical interviews, patients with AD scored significantly higher than healthy participants on five of the six subscores of the CDR (subscore *personal care* is the exception) as well as on the sum of boxes score. In the neuropsychological assessment, patients with AD performed significantly poorer than healthy participants on all neuropsychological tests except for Drawings. During the first ride in the driving simulator, patients with AD swerved (indicated by SDLP) significantly more and drove significantly slower when they were in a hurry than healthy participants. During Intersections a, the deviance from the speed limit differed between the groups and again patients with AD were driving slower than healthy participants. Pairwise comparisons are presented in Table 2. The pass rates on the on-road assessment also differed between patients with AD and healthy participants (χ^2^(2) = 28.22, p < .001). In the group of patients with AD, 35 (43.2%) patients passed the on-road assessment, 5 (6.2%) patients had a doubtful outcome and 41 (50.6%) patients failed. In the healthy comparison group, 40 (88.9%) participants passed the on-road assessment, 3 (6.7%) had a doubtful outcome and 2 (4.4%) failed the on-road assessment.

**Table 2:** Comparison of healthy participants with patients with Alzheimer’s disease (AD) on clinical interviews, neuropsychological assessment, and driving simulator rides.

**Clinical interviews**	Healthy (n = 43)	AD (n = 73)	F	p ^a^	ES ^b^
**Clinical Dementia Rating**					
Memory	0.035±0.129	0.801±0.462	112.8	< .001 *	2.04
Orientation	0.023±0.153	0.719±0.527	71.2	< .001 *	1.62
Judgement & Problem solving	0.023±0.107	0.630±0.493	63.2	< .001 *	1.53
Community affairs	0.000±0.000	0.356±0.282	68.2	< .001 *	1.59
Home & Hobbies	0.000±0.000	0.336±0.364	36.5	< .001 *	1.16
Personal care	0.000±0.000	0.034±0.174	1.672	.199	0.25
Sum of boxes	0.081±0.326	2.877±1.565	133.3	< .001 *	2.22
**Heteroanamnesis**					
Judgement driving safety	1.07±0.34	1.27±0.51	5.5	.021	0.44
Opinion cease driving	1.00±0.00	1.21±0.53	6.5	.012	0.50
**Anamnesis**					
Judgement driving safety	1.07±0.26	1.08±0.28	0.1	.811	0.04
Opinion cease driving	1.00±0.00	1.01±0.12	0.6	.445	0.11
**Driving questionnaire**					
Mean score	0.37±0.30	0.27±0.28	3.1	.079	0.34
Recent driving experience	2.98±1.10	2.62±1.05	3.1	.082	0.34
**Neuropsychological assessment**	Healthy (n = 45)	AD (n = 76)	F	p ^a^	ES ^b^
**MMSE**					
Total score	28.82±1.093	23.42±3.634	94.1	< .001 *	1.82
**TMT**					
TMT B-TMT A (sec)	63.33±33.20	164.49±88.14	54.5	< .001 *	1.39
**Drawings**					
Total score	6.40±1.24	5.91±1.69	2.9	.091	0.32
**Mazes**					
Maze 1, time (sec)	5.24±2.24	16.12±17.95	16.3	< .001 *	0.76
Maze 2, time (sec)	16.58±7.27	37.39±29.92	21.0	< .001 *	0.86
**RT S1**					
Reaction time (msec)	303.76±59.93	399.28±141.61	18.5	< .001 *	0.81
Motor time (msec)	241.38±67.05	302.91±108.89	11.7	.001 *	0.64
Variability in reaction time	42.40±21.25	73.84±67.64	9.2	.003 *	0.57
Variability in motor time	39.47±16.57	52.97±27.24	9.1	.003 *	0.57
**RT S2**					
Reaction time (msec)	257.22±55.34	322.24±121.01	11.5	.001 *	0.64
Motor time (msec)	211.24±52.65	253.20±101.18	6.7	.011 *	0.49
Variability in reaction time	39.84±14.87	63.78±49.45	10.0	.002 *	0.59
Variability in motor time	28.42±10.25	39.01±21.32	9.7	.002 *	0.59
**RT S3**					
Reaction time (msec)	514.24±92.30	710.08±288.92	19.4	< .001 *	0.83
Motor time (msec)	266.89±71.53	336.55±156.27	7.9	.006 *	0.53
Variability in reaction time	84.33±24.66	156.70±100.65	22.4	< .001 *	0.89
Variability in motor time	36.89±14.78	59.09±47.24	9.4	.003 *	0.58
**ATAVT**					
Performance parameter	-5.712±9.532	-15.475±11.431	23.2	< .001 *	0.91
**Hazard perception**					
Response time (sec)	4.36±0.64	5.51±0.78	69.7	< .001 *	1.57
Correct trials	15.76±2.08	13.47±3.20	18.3	< .001 *	0.81
**Traffic theory**					
Response time (sec)	6.42±1.09	7.67±1.06	37.8	< .001 *	1.16
Correct trials	21.20±3.40	18.09±3.24	25.1	< .001 *	0.94
**Driving simulator rides**	Healthy (n = 22)	AD (n = 56)	F	p ^a^	ES ^b^
**Lane tracking ride**					
Speed of choice (km/h)	79.05±6.65	70.92±12.79	8.0	.006	0.71
SDLP (cm)	21.26±6.33	27.81±8.18	11.4	.001 *	0.85
Speed in hurry (km/h)	90.04±8.26	80.31±12.50	11.3	.001 *	0.85
SDLP in hurry (cm)	23.00±6.51	27.06±7.70	4.8	.032	0.55
Number of collisions	0.0±0.0	0.0±0.0	NA	NA	NA
**Intersections a**					
Minimum speed Int 1 (km/h)	14.36±22.67	12.81±18.37	0.1	.756	0.08
Minimum speed Int 2 (km/h)	10.78±19.05	14.27±22.40	0.4	.521	0.16
Minimum speed Int 3 (km/h)	8.01±15.96	10.61±17.75	0.4	.551	0.15
Dev from speed limit (km/h)	-3.42±3.81	-8.85±7.30	10.9	.001 *	0.84
RT traffic lights (sec)	1.47±0.94	2.38±2.08	3.8	.055	0.49
Braking for car that pulls out	0.41±0.50	0.52±0.50	0.7	.394	0.22
Number of collisions	0.82±0.91	0.55±0.78	1.6	.204	0.33
**Intersections b**					
Minimum speed Int 1 (km/h)	3.56±5.84	13.17±19.10	5.3	.024	0.58
Minimum speed Int 2 (km/h)	0.63±1.24	19.24±30.35	8.2	.005	0.72
Minimum speed Int 3 (km/h)	4.57±10.09	18.19±23.24	7.0	.010	0.67
Dev from speed limit (km/h)	-3.19±4.55	-7.34±8.56	4.6	.035	0.55
RT traffic lights (sec)	0.96±0.66	2.11±1.77	8.7	.004	0.74
Braking for car that pulls out	0.50±0.51	0.52±0.50	0.0	.889	0.04
Number of collisions	0.36±0.58	0.38±0.59	0.0	.939	0.03
**Merging ride**					
Speed while merging (km/h)	85.75±13.74	84.11±13.71	0.2	.636	0.12
Deceleration rear car (km/h)	-1.10±2.00	-1.10±1.77	0.0	.999	0.00
Time headway merging (sec)	1.26±0.79	1.13±0.67	0.5	.472	0.18
Minimum time headway (sec)	0.33±0.33	0.41±0.36	0.9	.338	0.24

^a^ Statistical significance is indicated by *. Significance level of .05 was corrected using the Bonferroni-Holm procedure.

^b^ Effect size (ES) is indicated by Cohen’s d.

Abbreviations: MMSE, Mini Mental State Examination; TMT, Trailmaking Test; RT, Reaction time; ATAVT, Adaptive Tachistoscopic Traffic Perception Test; SDLP, standard deviation of lateral position; NA, not applicable; Int, intersection with need to give right of way; Dev, deviance.

### Fitness to drive of people with AD

The patients with AD were divided into a pass and a fail group. The *pass group* entailed the 35 (43.2%) patients who passed the on-road assessment. The *fail group* included the other 46 (56.8%) patients and included both the patients who had a doubtful outcome and the patients who failed the on-road assessment. MANOVA revealed significant differences between the *pass* and *fail group* with regard to clinical interviews (Wilk’s lambda = 0.655, F(12,60) = 2.635, p = .007, η^2^ = .345), neuropsychological assessment (Wilk’s lambda = 0.426, F(23,52) = 3.047, p < .001, η^2^ = .574), and driving simulator rides (Wilk’s lambda = 0.419, F(22,33) = 2.077, p = .028, η^2^ = .581). In the clinical interviews, the *fail group* scored significantly higher on the CDR subscore *judgement & problem solving* as well as on the sum of boxes score than the *pass group*. Moreover, the *fail group* had driven less kilometres in the past twelve months (indicated by recent driving experience) than the *pass group*. The *fail group* performed significantly worse than the *pass group* on seven neuropsychological tests, including the MMSE, Drawings, Maze 2, RT S1, RT S2, Hazard perception and Traffic theory. In the driving simulator rides, there was one significant difference showing that in the *fail group* the car driving behind the patient needed to slow down much more to avoid a collision after the patient merged on the motorway than in the *pass group*. Pairwise comparisons are presented in Table 3.

**Table 3:** Comparison of patients with Alzheimer’s disease (AD) who pass and patients with AD who fail the on-road test on clinical interviews, neuropsychological assessment, and driving simulator rides.

**Clinical interviews**	Pass (n = 35)	Fail (n = 38)	F	p ^a^	ES ^b^
**Clinical Dementia Rating**					
Memory	0.671±0.382	0.921±0.500	5.6	.020	0.56
Orientation	0.543±0.460	0.882±0.538	8.2	.005	0.68
Judgement & Problem solving	0.400±0.292	0.842±0.547	18.1	< .001 *	1.00
Community affairs	0.286±0.251	0.421±0.297	4.4	.040	0.49
Home & Hobbies	0.271±0.329	0.395±0.388	2.1	.149	0.34
Personal care	0.014±0.085	0.053±0.226	0.9	.349	0.23
Sum of boxes	2.186±0.971	3.513±1.742	15.8	< .001 *	0.93
**Heteroanamnesis**					
Judgement driving safety	1.17±0.38	1.37±0.59	2.8	.098	0.40
Opinion cease driving	1.09±0.37	1.32±0.62	3.6	.061	0.45
**Anamnesis**					
Judgement driving safety	1.03±0.17	1.13±0.34	2.6	.112	0.37
Opinion cease driving	1.03±0.17	1.00±0.00	1.1	.301	0.26
**Driving questionnaire**					
Mean score	0.24±0.22	0.31±0.32	1.1	.292	0.25
Recent driving experience	3.03±1.04	2.24±0.91	12.0	.001 *	0.81
**Neuropsychological assessment**	Pass (n = 35)	Fail (n = 41)	F	p ^a^	ES ^b^
**MMSE**					
Total score	25.11±2.23	21.98±3.98	17.1	< .001 *	0.95
**TMT**					
TMT B-TMT A (sec)	144.51±90.02	181.54±83.85	3.4	.068	0.43
**Drawings**					
Total score	6.69±1.29	5.24±1.71	16.7	< .001 *	0.94
**Mazes**					
Maze 1, time (sec)	12.17±16.29	19.49±18.80	3.2	.076	0.41
Maze 2, time (sec)	26.40±22.43	46.78±32.48	9.8	.003 *	0.72
**RT S1**					
Reaction time (msec)	350.69±84.33	440.76±166.52	8.4	.005	0.67
Motor time (msec)	260.54±74.28	339.07±120.96	11.2	.001 *	0.77
Variability in reaction time	57.69±29.15	87.63±86.21	3.8	.054	0.45
Variability in motor time	49.77±21.32	55.71±31.44	0.9	.347	0.22
**RT S2**					
Reaction time (msec)	272.74±51.21	364.49±145.79	12.5	.001 *	0.81
Motor time (msec)	208.94±68.28	290.98±109.79	14.7	< .001 *	0.88
Variability in reaction time	48.69±20.33	76.66±62.16	6.5	.013	0.59
Variability in motor time	32.77±15.90	44.34±23.96	5.9	.017	0.56
**RT S3**					
Reaction time (msec)	620.51±231.82	786.54±312.77	6.7	.012	0.60
Motor time (msec)	283.34±127.09	381.98±165.73	8.2	.005	0.66
Variability in reaction time	132.34±84.65	177.49±109.28	3.9	.051	0.46
Variability in motor time	44.49±34.09	71.56±53.40	6.7	.012	0.59
**ATAVT**					
Performance parameter	-13.181±10.500	-17.433±11.948	2.7	.106	0.38
**Hazard perception**					
Response time	5.30±0.71	5.69±0.80	5.1	.027	0.52
Correct trials	15.20±2.87	12.00±2.71	24.9	< .001 *	1.15
**Traffic theory**					
Response time	7.18±0.94	8.08±1.00	16.0	< .001 *	0.92
Correct trials	19.11±2.64	17.22±3.48	7.0	.010	0.61
**Driving simulator rides**	Pass (n = 28)	Fail (n = 28)	F	p ^a^	ES ^b^
**Lane tracking ride**					
Speed of choice (km/h)	71.07±10.66	70.78±14.82	0.0	.932	0.02
SDLP (cm)	26.56±6.49	29.05±9.53	1.3	.257	0.31
Speed in hurry (km/h)	81.35±12.25	79.27±12.89	0.3	.540	0.17
SDLP in hurry (cm)	25.60±5.94	28.51±9.01	2.0	.159	0.51
Number of collisions	0.0±0.0	0.0±0.0	NA	NA	NA
**Intersections a**					
Minimum speed Int 1 (km/h)	11.05±15.77	14.57±20.80	0.5	.478	0.19
Minimum speed Int 2 (km/h)	6.89±17.04	21.66±24.87	6.7	.012	0.69
Minimum speed Int 3 (km/h)	7.88±15.09	13.35±19.96	1.3	.252	0.31
Dev from speed limit (km/h)	-8.50±6.14	-9.20±8.40	1.1	.722	0.10
RT traffic lights (sec)	1.71±1.59	3.04±2.32	6.3	.015	0.67
Braking for car that pulls out	0.57±0.50	0.46±0.51	0.6	.432	0.22
Number of collisions	0.32±0.55	0.79±0.92	5.3	.025	0.62
**Intersections b**					
Minimum speed Int 1 (km/h)	10.93±16.27	15.42±21.63	0.8	.385	0.23
Minimum speed Int 2 (km/h)	10.24±24.02	28.25±33.62	5.3	.025	0.62
Minimum speed Int 3 (km/h)	12.98±19.43	23.40±25.82	2.9	.093	0.46
Dev from speed limit (km/h)	-7.47±7.71	-7.21±9.48	0.0	.909	0.03
RT traffic lights (sec)	1.51±1.56	2.71±1.80	7.1	.010	0.71
Braking for car that pulls out	0.68±0.48	0.36±0.49	6.2	.016	0.66
Number of collisions	0.25±0.52	0.50±0.64	2.6	.113	0.43
**Merging ride**					
Speed while merging (km/h)	88.00±12.32	80.22±14.13	4.8	.032	0.59
Deceleration rear car (km/h)	-0.33±0.79	-1.87±2.13	12.9	.001 *	0.96
Time headway merging (sec)	0.90±0.60	1.36±0.66	7.5	.008	0.73
Minimum time headway (sec)	0.33±0.18	0.50±0.47	3.4	.072	0.49

^a^ Statistical significance is indicated by *. Significance level of .05 was corrected using the Bonferroni-Holm procedure.

^b^ Effect size (ES) is indicated by Cohen’s d.

Abbreviations: MMSE, Mini Mental State Examination; TMT, Trailmaking Test; RT, Reaction time; ATAVT, Adaptive Tachistoscopic Traffic Perception Test; SDLP, standard deviation of lateral position; NA, not applicable; Int, intersection with need to give right of way; Dev, deviance.

### Prediction of fitness to drive of patients with AD

#### Prediction of fitness to drive using clinical interviews

First, we examined which independent variables (possible predictor variables) from the clinical interviews were correlating (α = .05) with FITtoDRIVE. Variables of the CDR that correlated significantly with FITtoDRIVE were subscores *memory* (r = -.259, p = .020), *orientation* (r = -.313, p = .004), *judgement & problem solving* (r = -.414, p < .001) and *community affairs* (r = -.253, p = .023) as well as the sum of boxes score (r = -.417, p < .001). Furthermore, significant correlations with FITtoDRIVE were found for the informant’s opinion whether the patient should cease driving (r = -.273, p = .015), for the patient’s judgement of one’s own driving safety (r = -.229, p = .040) and recent driving experience (r = .424, p < .001). This selection of variables was entered to a forward binary logistic regression analysis to determine the validity of the predictor variables in predicting FITtoDRIVE. A significant model was found to predict FITtoDRIVE, χ^2^(4, N = 79) = 31.604, p < .001, on the basis of CDR subscores *orientation* and *judgement & problem solving*, the patient’s own judgement about driving safety and recent driving experience. The model explained 33.0% of the total variance (Cox & Snell R^2^) and classified 73.4% of the patients correctly as fit or unfit to drive. A summary of the forward logistic regression analysis with the predictor variables from the clinical interviews is presented in Table 4. Subsequently, a DFA was conducted on the basis of the variables suggested by the logistic regression predicting FITtoDRIVE. A significant model emerged (Wilk’s lambda = 0.673, χ^2^(4) = 30.486, p < .001, r = .572). A prediction equation was generated by summating predictor variables weighted with their unstandardized canonical discriminant coefficients: CDR *orientation* x 0.675 + CDR *judgement & problem solving* x 1.036 + Judgement of patient about driving safety x 1.250 + Recent driving experience x -0.576. With this prediction equation, a new predictor variable for the clinical interviews was created. The accuracy of the predictor variable for the clinical interviews in detecting patients who are unfit to drive (n = 46) relative to patients who are fit to drive (n = 35) was examined by means of an ROC analysis. A significantly higher accuracy than chance of detecting patients who are unfit to drive was revealed (AUC = .835, SE = 0.044, p < .001).

**Table 4:** Summary of forward logistic regression analyses of predictor variables from the clinical interviews, neuropsychological assessment and driving simulator rides on fitness to drive for patients with Alzheimer’s disease.

Predictor variables	B	*SE* B	Wald	p	Odds ratio
**Clinical interviews**					
*Model*					
CDR Orientation	-1.088	-0.603	3.254	.071	0.337
CDR Judgement & Problem solving	-1.840	0.856	4.622	.032	0.159
Anamnesis Judgement driving safety	-1.470	1.153	1.627	.202	0.230
Recent driving experience	0.710	0.293	5.887	.015	2.035
Total R^2^ = 0.330*					
*Excluded variables*			Score		
CDR Memory			0.324	.569	
CDR Community affairs			1.531	.216	
CDR Sum of boxes score			0.109	.742	
Heteroanamnesis Opinion cease driving			0.429	.513	
**Neuropsychological assessment**					
*Model*					
MMSE	0.277	0.118	5.536	.019	1.319
RT S2 RT	-0.011	0.005	4.734	.030	0.989
Hazard perception Correct trials	0.411	0.138	8.903	.003	1.508
Traffic theory Response time	-0.493	0.334	2.174	.140	0.611
Total R^2^ = 0.444*					
*Excluded variables*			Score		
TMT B-TMT A			0.091	.763	
Drawings			1.582	.208	
Maze 2 time			0.323	.570	
RT S1 RT			0.013	.910	
RT S1 MT			1.265	.261	
RT S1 Variability in RT			0.788	.375	
RT S2 MT			1.709	.191	
RT S2 Variability in RT			3.439	.064	
RT S2 Variability in MT			0.045	.831	
RT S3 RT			0.029	.865	
RT S3 MT			0.233	.629	
RT S3 Variability in RT			0.018	.892	
RT S3 Variability in MT			0.376	.540	
Hazard perception Response time			2.284	.131	
Traffic theory Correct trials			1.000	.317	
**Driving simulator rides**					
*Model*					
Minimum speed at intersection 2 in Int a	-0.030	0.017	3.159	.075	0.970
Number of collisions in Int a	-1.066	0.494	4.656	.031	0.345
Deceleration of rear car after merging	0.679	0.333	4.163	.041	1.971
Time headway after merging	-0.991	0.637	2.421	.120	0.371
Total R^2^ = 0.377*					
*Excluded variables*			Score		
RT traffic lights in Int a			0.223	.637	
Minimum speed at intersection 2 in Int b			0.003	.959	
RT traffic lights in Int b			0.468	.494	
Braking for car that pulls out in Int b			1.885	.170	
Speed while merging			2.024	.155	

Abbreviations: MMSE, Mini Mental State Examination; TMT, Trailmaking Test; RT, Reaction time; MT, Motor time; Int a, first intersections ride; Int b, second intersections ride;

* Cox & Snell R^2^.

#### Prediction of fitness to drive using neuropsychological assessment

Variables of paper-and-pencil tests that correlated significantly with FITtoDRIVE were the MMSE (r = .459, p < .001), TMT B-TMT A (r = -.230, p = .039), Drawings (r = .439, p < .001) and Maze 2 (r = -.333, p = .003). Of the RT tests, all variables describing RT (RT S1: r = -.309, p = .005; RT S2: r = -.369, p = .001; RT S3: r = -.282, p = .012), MT (RT S1: r = -.342, p = .002; RT S2: r = -.399, p < .001; RT S3: r = -.294, p = .008) and variability in RT (RT S1: r = -.222, p = .047; RT S2: r = -.287, p = .009; RT S3: r = -.234, p = .038) and variability in MT (RT S2: r = -.250, p = .024; RT S3: r = -.266, p = .018) correlated with FITtoDRIVE except the variability in MT of RT S1. Furthermore, significant correlations with FITtoDRIVE were found for the response time (r = -.265, p = .017) and the number of correct trials (r = .510, p < .001) of the hazard perception test, as well as the response time (r = -.385, p < .001) and the number of correct trials (r = .269, p = .016) of the traffic theory test. Again, the correlating variables were used in a forward binary logistic regression analysis to determine the validity of the predictor variables in predicting FITtoDRIVE. A significant model was found to predict FITtoDRIVE, χ^2^(4, N = 77) = 45.149, p < .001, on the basis of the MMSE, RT S2 RT, correct trials of the hazard perception test and the response time in the traffic theory test. The model explained 44.4% of the total variance (Cox & Snell R^2^) and classified 81.8% of the patients correctly as fit or unfit to drive. A summary of the forward logistic regression analysis with the predictor variables from the neuropsychological assessment is presented in Table 4. Afterwards, a DFA was performed on the basis of the variables suggested by the logistic regression predicting FITtoDRIVE. The DFA resulted in a significant model (Wilk’s lambda = 0.601, χ^2^(4) = 38.717, p < .001, r = .632). A prediction equation was generated by summating predictor variables weighted with their unstandardized canonical discriminant coefficients: MMSE x 0.129 + RT S2 RT x -0.003 + Correct trials of Hazard perception x 0.206 + Response time of Traffic theory x -0.310. The accuracy of the predictor variable for neuropsychological assessment in detecting patients who are unfit to drive (n = 45) relative to patients who are fit to drive (n = 35) was examined using an ROC analysis. A significantly higher accuracy than chance of detecting patients who are unfit to drive was revealed (AUC = .905, SE = 0.035, p < .001).

#### Prediction of fitness to drive using driving simulator rides

Variables of the driving simulator rides that correlated significantly with FITtoDRIVE were in Intersections a Minimum speed Int 2 (r = -.333, p = .012), RT traffic lights (r = -.323, p = .015) and Number of collisions (r = -.299, p = .025), in Intersections b Minimum speed at Int 2 (r = -.318, p = .016), RT traffic lights (r = -.358, p = .006) and Braking for car that pulls out (r = .334, p = .011) and in the Merging ride, Speed while merging (r = .286, p = .031), Deceleration rear car (r = .446, p = .001) and Time headway merging (r = -.367, p = .005). With these variables, a forward binary logistic regression analysis was performed to determine the validity of the predictor variables in predicting FITtoDRIVE. A significant model was found to predict FITtoDRIVE, χ^2^(4, N = 56) = 26.461, p < .001, on the basis of Minimum speed Int 2 (with high values indicating unsafe driving) and Number of collisions in Intersections a, Deceleration rear car and Time headway merging in the Merging ride. The model explained 37.7% of the total variance (Cox & Snell R^2^) and classified 75.0% of the patients correctly as fit or unfit to drive. A summary of the forward logistic regression analysis with the predictor variables from the driving simulator rides is presented in Table 4. Successively, a DFA was conducted based on the variables included by the regression model to predict FITtoDRIVE. Using DFA, a significant model was found (Wilk’s lambda = 0.617, χ^2^(4) = 25.151, p < .001, r = .619). A prediction equation was generated by summating predictor variables weighted with their unstandardized canonical discriminant coefficients: Minimum speed Int 2 in Intersections a x 0.021 + Number of collisions in Intersections a x 0.738 + Deceleration rear car in the Merging ride x -0.367 + Time headway merging in the Merging ride x 0.732. The accuracy of the predictor variable for driving simulator rides in detecting patients who are unfit to drive (n = 28) relative to patients who are fit to drive (n = 28) was examined by an ROC analysis. A significantly higher accuracy than chance of detecting patients who are unfit to drive was revealed (AUC = .861, SE = 0.047, p < .001).

#### Comparing prediction models of fitness to drive

**Fig 1:**
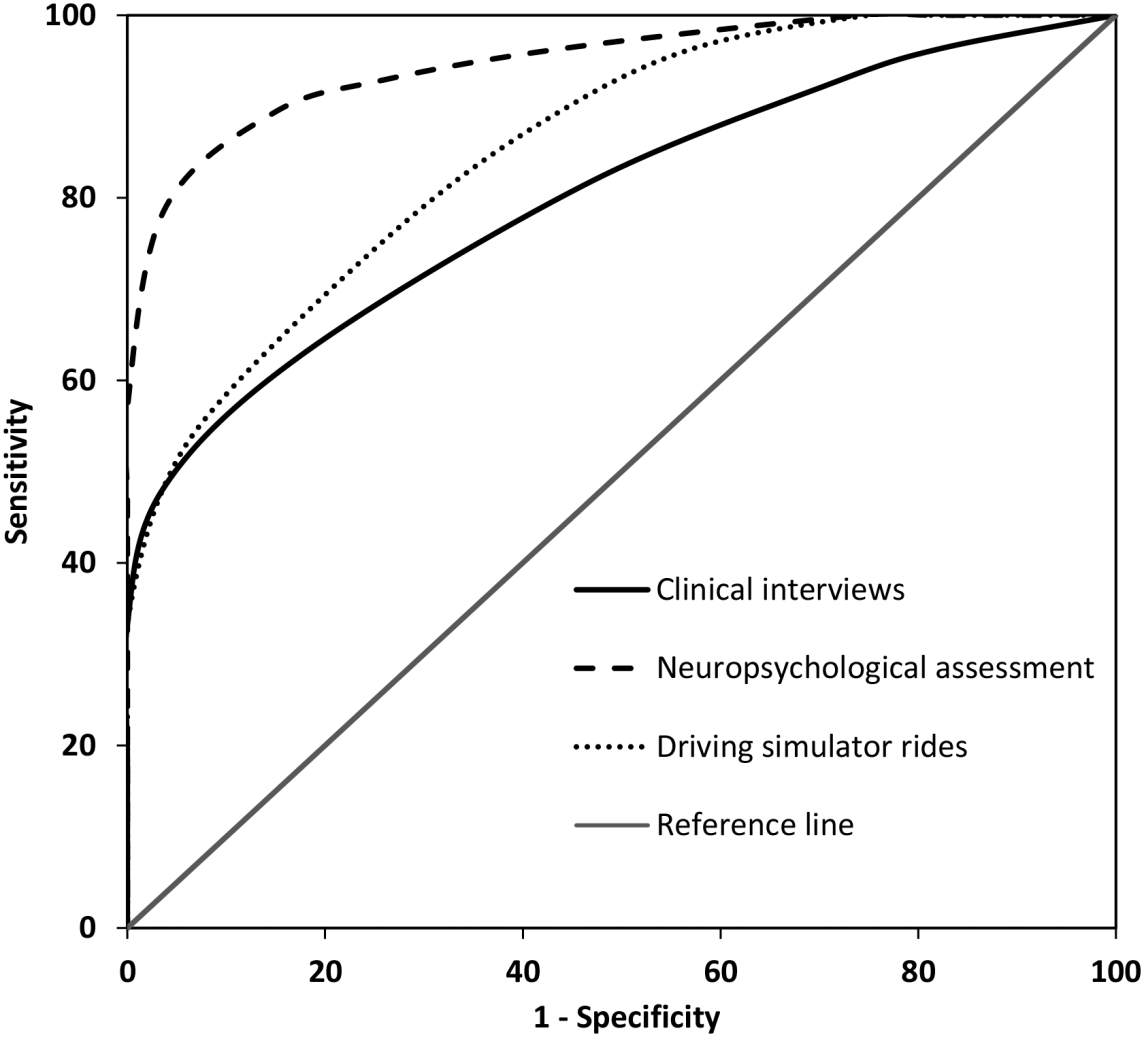
ROC curves of the predictor variables of the clinical interviews, neuropsychological assessment and driving simulator rides.

In order to compare the three prediction models in their accuracy to identify patients who are unfit to drive, patients with complete data on all three types of assessments were selected (n = 55). The accuracy of the predictor variables for clinical interviews, neuropsychological assessment and driving simulator rides in detecting patients who are unfit to drive (n = 27) relative to patients who are fit to drive (n = 28) were examined by ROC analyses. Though all predictor variables add significant accuracy compared to prediction on chance level, a comparison of prediction accuracies demonstrated that neuropsychological assessment was the best in identifying patients unfit to drive (AUC = .946, SE = 0.029, p < .001), followed by driving simulator rides (AUC = .856, SE = 0.049, p < .001), and clinical interviews (AUC = .796, SE = 0.060, p < .001). A visual comparison of ROC curves for the three prediction models is presented in Fig 1.

#### Combining prediction models of fitness to drive

**Fig 2:**
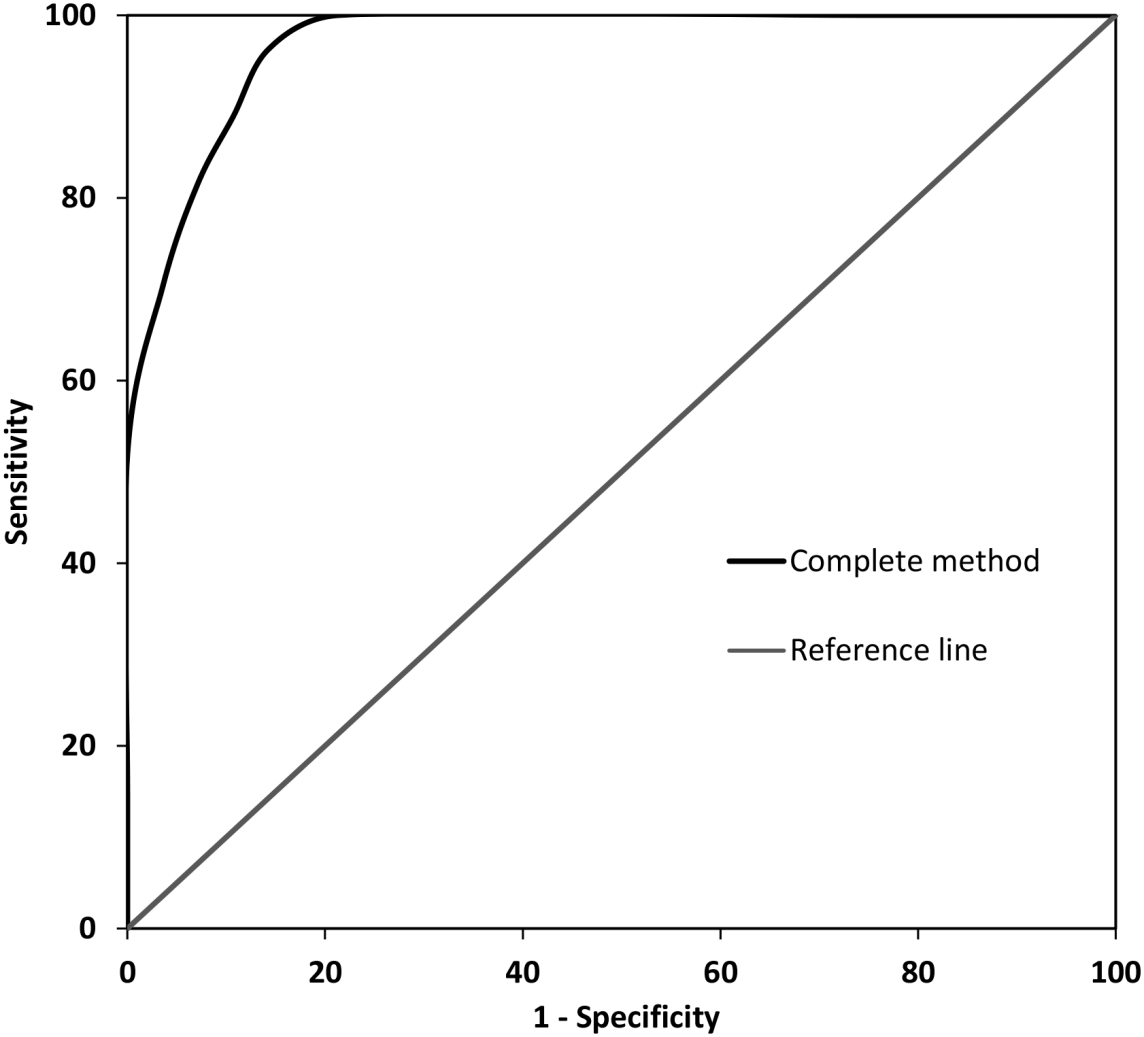
ROC curve of the predictor variable of the complete method.

The three predictor variables were entered in a hierarchical binary logistic regression analysis to investigate whether using more than one type of assessment improves the predictive accuracy in detecting patients being unfit to drive. According to the common procedure in clinical practice, the predictor variable of clinical interviews was entered in block 1 (χ^2^(1, N = 55) = 41.110, p < .001), the predictor variable of neuropsychological assessment was entered in block 2 (χ^2^(2, N = 55) = 48.527, p < .001) and the predictor variable of driving simulator rides was entered in block 3 (χ^2^(3, N = 55) = 52.477, p < .001). The model increased significantly in predictive validity with each step, i.e. the model of block 1 explained 52.6% of the total variance (Cox & Snell R^2^), the model of block 2 added 6.0% to the explained variance (Cox & Snell R^2^ = 58.6%) and the model of block 3 added another 2.9% to the explained variance (Cox & Snell R^2^ = 61.5%). With the model of block 3, 87.3% of the patients were correctly classified as fit or unfit to drive. Subsequently, a DFA was conducted with all three predictor variables and FITtoDRIVE, which resulted in a significant model (Wilk’s lambda = 0.412, χ^2^(3) = 45.720, p < .001, r = .767). A prediction equation was generated by summating all variables weighted by their unstandardized canonical discriminant coefficients: Clinical interviews x 0.328 + Neuropsychological assessment x -0.620 + Driving simulator rides x 0.483. With this prediction equation, a final predictor variable was created representing the complete method. The accuracy of this predictor variable in detecting patients being unfit to drive (n = 27) relative to patients being fit to drive (n = 28) was examined by means of an ROC analysis. A high accuracy of detecting patients who are unfit to drive was revealed (AUC = .974, SE = 0.018, p < .001). A graphical plot of the ROC curve representing the final predictor variable is shown in Fig 2.

In order to determine a suitable cut-off for the final predictor variable, a range of cut-offs were explored in terms of their classification accuracies, including sensitivity, specificity, positive predictive value (PPV) and negative predictive value (NVP) (Table 5). When aiming for the highest possible combination of sensitivity and specificity (as indicated by Youden’s index [57]), the best cut-off is -0.6. Applying this cut-off, 51 out of 55 patients were classified correctly, corresponding to a predictive accuracy of 92.7%, with 3 patients incorrectly classified as failing (false negative) and 1 patient incorrectly classified as passing (false positive) (Table 6). However, one could also argue that it has highest priority to correctly identify patients who are unfit to drive, resulting in a best cut-off of -0.8. With this cut-off, 50 out of 55 patients were classified correctly, corresponding to a predictive accuracy of 90.9%, with 5 patients incorrectly classified as failing (false negative), but no patients incorrectly classified as passing (false positive) (Table 6).

**Table 5:** Classification accuracy of the final predictor variable (including clinical interviews, neuropsychological assessment and driving simulator rides) in detecting patients being unfit to drive (n = 27) relative to patients being fit to drive (n = 28).

Cut-off final predictor variable	Sensitivity	Specificity	Youden’s index	PPV	NPV
-0.8	100.0	82.1	0.821	84.4	100.0
-0.7	96.3	85.7	0.820	86.7	96.0
-0.6	96.3	89.3	0.856	89.7	96.2
-0.5	88.9	89.3	0.782	88.9	89.3
-0.4	88.9	92.9	0.818	92.3	89.7
-0.3	81.5	92.9	0.744	91.7	83.9
-0.2	81.5	92.9	0.744	91.7	83.9
-0.1	81.5	92.9	0.744	91.7	83.9
0.0	77.8	92.9	0.707	91.3	81.3
0.1	74.1	92.9	0.670	90.9	78.8
0.2	70.4	92.9	0.633	90.5	76.5

Abbreviations: PPV, positive predictive value; NPV, negative predictive value

**Table 6:** Predictive accuracy of the final predictor variable with cut-off -0.6 (left) and -0.8 (right).

Prediction (-0.6)	Fitness to drive			Prediction (-0.8)	Fitness to drive		
	**Fail**	**Pass**	**Total**		**Fail**	**Pass**	**Total**
**Fail**	26	3	29	**Fail**	27	5	32
**Pass**	1	25	26	**Pass**	0	23	23
**Total**	27	28	55	**Total**	27	28	55

## Discussion

The present study aimed to develop a method for the prediction of FTDr in patients with AD which can be applied in a clinical setting, including clinical interviews, neuropsychological assessment and driving simulator rides. The criterion FITtoDRIVE was the binary outcome of an on-road driving assessment.

Patients with AD performed significantly worse than a healthy comparison group on the clinical interviews, neuropsychological assessment and driving simulator rides reaching effect sizes of up to 2.22 (Cohen’s d) (Table 2). Additionally, the percentage of patients with AD who failed the on-road assessment (56.8%) was considerably larger than the percentage of healthy participants who failed the on-road assessment (11.1%). Since the vast majority of healthy participants passed the on-road assessment, age alone does not seem to indicate unsafe driving [58,59]. The healthy comparison group was not included in further analyses, because the key objective was to differentiate between fit and unfit drivers with AD.

Approximately half of the patients with AD passed the on-road assessment and the other half failed, indicating that AD affects driving. Correspondingly, patients with AD were consistently found to represent an at-risk group for unsafe driving in previous research [5,8–11]. Despite the association with unsafe driving, half of the patients with AD were evaluated as fit to drive in the on-road assessment. The finding that some but not all patients with AD are unfit to drive is in line with previous studies [9–11] and supports the need for the investigation of FTDr in this population.

Within the group of patients with AD, patients who failed the on-road assessment performed significantly worse than patients who passed the on-road assessment with regard to clinical interviews, neuropsychological assessment and driving simulator rides reaching effect sizes of up to 1.15 (Cohen’s d) (Table 3). The first goal was to determine which combination of measures from each type of assessment is most predictive of failing the on-road assessment. Clinical interviews were shown to be useful for the prediction of FITtoDRIVE with an overall accuracy of 83.5% and 33.0% explained variance. Two sub-scores of the CDR, i.e. *orientation* and *judgement & problem solving*, were shown to significantly predict FITtoDRIVE. Impairments in orientation may lead to failure to perceive all relevant aspects of the infrastructure and to an inability to follow routes [12]. Impairments in judgement could result in strategic and tactical errors, such as driving in adverse weather conditions and overtaking in dangerous circumstances. Although CDR subscore *memory* correlated with FITtoDRIVE in bivariate analysis, the predictive accuracy of the logistic regression did not improve when entering this variable to the model. The patient’s own judgement about driving safety and recent driving experience were the two predictor variables that significantly contribute to the prediction model. These two findings suggest that patients with mild AD may have meta-analytic skills and are able to evaluate their own abilities and functioning, in contradiction to previous research showing impaired awareness of their functioning in activities of daily living [60]. The self-evaluation of driving abilities might have also affected recent driving experience, i.e. patients who evaluate their own driving abilities as limited may reduce their driving experience [61].

Neuropsychological test performance was also demonstrated to be predictive for FITtoDRIVE with an overall accuracy of 90.5% and 44.4% explained variance. One of the four predictors was the MMSE, which supports the assumption that patients with a more severe dementia are less likely to drive safely [14,15]. However, the MMSE alone has previously been found to be insufficient for predicting FTDr [59]. Another predictor included in the model was a reaction time measure of a selective auditory attention test. Slow reaction times of participants may lead to too slow responses to traffic situations on the road as well. Two additional measures were included in the model that do not come from classical neuropsychological tests, but from tests specifically developed for the driving context, i.e. the correct trials of the hazard perception test and the response time in the traffic theory test. The knowledge about traffic rules and recognition of hazards and selection of appropriate actions according to these hazards are important aspects for safe driving so that it is not surprising that these measures contribute to the prediction of FTDr. Further inspection of the predictors derived from the neuropsychological assessment shows that the MMSE may be available from a routine clinical evaluation and that simple reaction time tests are rather common in clinical neuropsychological assessments. However, a hazard perception test and traffic theory test are tests specifically designed for the assessment of FTDr. Dobbs and colleagues (2002) [21] have suggested that tests explicitly designed for the prediction of FTDr may be much stronger predictors than classical neuropsychological tests. The present results support this view only partially, since two predictors originate from specialized traffic tests while two other predictors are derived from classical neuropsychological tests. When looking at the odds ratios of the logistic regression analysis from the neuropsychological assessment (Table 4), it appears that the hazard perception test is very important for the prediction of FITtoDRIVE with 50.8% change in likelihood of FITtoDRIVE per one unit change. A substantially effect on FITtoDRIVE is also revealed for the MMSE, with an odds ratio indicating a 31.9% increase in likelihood of FITtoDRIVE per one unit change. This implicates that classical neuropsychological tests and specialized traffic tests both can make an important contribution to the prediction of FTDr.

Driving simulator rides significantly predict FITtoDRIVE in patients with AD as well, with an overall accuracy of 86.1% and 37.7% explained variance. Two predictor variables included in the model were measured during the first intersections ride, the lowest speed when approaching a specific intersection and the number of collisions. The other two predictor variables were measured during merging, i.e. the deceleration of the rear car after merging and the time headway to the car in front after merging. These predictor variables indicate that patients who drive fast towards an intersection where they have to give way, but drive slow when they merge on the motorway right in front of another car, are likely to fail the on-road assessment. These findings may indicate that patients who are unfit to drive do not anticipate for upcoming traffic situations, since they do not make appropriate speed adjustments. The driving simulator results also suggest that it is important for the prediction of FTDr to look at behaviour at intersections where the participant has to give way and a merging manoeuvre besides measuring the number of collisions, in particular as collisions are high impact events that should be avoided in driving simulator research. Decreased lateral position control (SDLP) is frequently used for studying the effects of alcohol and drugs on driving behaviour [62,63], but was not found to predict FITtoDRIVE in patients with AD.

The second goal of the present study was to compare the predictive value of the three types of assessments. When comparing the accuracy of the prediction models on the same sample of patients with AD (n = 55), neuropsychological assessment provided the best prediction of FITtoDRIVE (94.6% accuracy), followed by driving simulator rides (85.6% accuracy) and clinical interviews (79.6% accuracy) (Fig 1). A clinical interview may not always be very informative, because it requires meta-analytic skills and self-evaluation from the patient. Although results presented above demonstrated these skills appear to be preserved at mild stages of AD, previous research showed that AD may affect these skills in the course of the disease [60]. Furthermore, it can be difficult to find an informant, which is crucial for a reliable prediction of FTDr on the basis of clinical interviews. As a result, clinical interviews are less objective and less standardized than neuropsychological tests and driving simulator rides. Nevertheless, driving simulator rides were also not sufficiently predictive if used alone. Although driving simulator rides share many characteristics with on-road driving (high *face validity*), it has also been stressed that driving in a driving simulator is not the same as driving on the road [18,63]. Moreover, driving simulators are originally not designed as a clinical tool while neuropsychological tests are. This could explain why especially these tests are predictive for FTDr in patients with AD.

The third goal of the present study was to examine the best possible combination of the three types of assessments. A combination of clinical interviews, neuropsychological assessment and driving simulator rides presented the best prediction of FITtoDRIVE, yielding 97.4% accuracy and 61.5% explained variance. Hence, a more thorough assessment provides a better prediction model than the neuropsychological assessment only. When using all three types of assessments and a cut-off of -0.6, the recommendation of Kay and colleagues (2012) [19] to aim for both sensitivity and specificity of at least 90% is very close to being met (sensitivity 96.3%, specificity 89.3%). One could argue that all patients with AD who are unfit to drive should always be classified correctly, because patients with AD who are unfit to drive will probably remain unfit to drive due to the progressive course of AD, resulting in a recommended cut-off of -0.8. With cut-off -0.8, the sensitivity is 100% and the specificity is still high with 82.1%. Both cut-offs resulted in a classification accuracy above 90%. Previous studies already suggested that using multiple tests may help in the prediction of FTDr [30]. The present results suggest not only the use of multiple tests, but also the use of multiple types of assessments. The three types of assessments appear to provide non-redundant and different types of information that are all useful for the prediction of FTDr. The clinical interviews may provide information about recent functioning of the patient in daily life, while a neuropsychological assessment informs about specific cognitive abilities and driving simulator rides about operational and tactical driving skills.

### Limitations and future directions

Self-reports of patients with AD have limitations. A pitfall is asking patients with AD whether they think they should cease driving, because patients who participate in an FTDr assessment in general wish to continue driving. However, when asking whether they are driving less safely than when they were middle-aged, they might admit that this is the case. In the present study, both questions were asked. No patients answered that they think they should cease driving, but their own judgement of driving safety was found to be predictive for FITtoDRIVE. Recent driving experience is another important predictor. It may be difficult for patients with AD to estimate the kilometres driven in the preceding twelve months, but here informants may be able to assist.

Simulator sickness is a common problem of older drivers [32,33]. In the current study, countermeasures were used, i.e. simulator scenarios included no sharp turns and relatively few elements in the landscape, and the driving simulator had a high frame rate of 60 images per second [64]. Nevertheless, simulator sickness occurred in approximately a third of the participants. Such high rates of simulator sickness have been found in older drivers before [32,33]. Simulator sickness is very inconvenient for the driver and might influence motivation and driving performance. For this reason, all participants who reported symptoms of simulator sickness were excluded entirely from analyses involving driving simulator rides. Participants were not invited to complete the driving simulator rides at another time in order to prevent another experience with simulator sickness (ethical reason). Unfortunately, simulator sickness leads to many missing data. The high rate of simulator sickness in older drivers limits the clinical utility of the driving simulator for evaluations of older drivers. Moreover, a selection bias might emerge as it remains unknown whether simulator sickness affects individuals randomly, or whether it is associated with their ability to perform in the driving simulator. This might affect a prediction model derived from the driving simulator data. Although Mullen and colleagues (2010)[33] suggested that simulator sickness is not related to driving performance, patients with simulator sickness who failed the on-road assessment are overrepresented in the current study, i.e. 7 patients (29.2%) who suffered from simulator sickness passed the on-road assessment while 17 patients (70.8%) with simulator sickness failed the on-road assessment. The frequent occurrence of simulator sickness and the resulting selection bias are problems that have to be dealt with when implementing driving simulator rides for the evaluation of older drivers with AD in a clinical setting. Numerous attempts are being made to reduce the occurrence of simulator sickness, e.g. by optical corrections, initial acclimation or olfactory cues [65–67],. If these attempts will lead to a marked reduction in rates of simulator sickness, driving simulator rides will become a promising method for investigating FTDr.

Although the classification accuracies are very high with both suggested cut-offs, there are still several misclassifications which may lead to severe consequences for the individual and other road users. Patients who are classified as unfit to drive while they are not (false negatives) will be limited in independent mobility and autonomy for no reason [5,7]. Patients who are classified as fit to drive while they are not (false positives) will continue to drive unsafely which poses risks for both patients and public. As an explanation for false positives, it can be speculated that patients with AD who pass tests and measures for FTDr might still be impaired at on-road driving, as on-road driving requires the integration of several functions and abilities under real life circumstances, and may thus represent a more complex process in comparison to rather isolated tests and measures at the clinical assessment of FTDr. In future research, it would be interesting to evaluate whether more complex assessment methods that require integration of functions and skills could predict such failure on the road [68,69]. As an explanation of false negatives, it must be considered that patients with AD may be able to adapt to or compensate for their cognitive impairment when driving on the road, e.g. by very careful driving behaviour or much driving experience [59]. However, years of driving experience did not correlate with FTDr in this study. Importantly, it is possible that patients with AD who are misclassified as unfit to drive might become unfit to drive soon after the assessment because of their progressive disease, indicating that these patients with AD may be advised to cease driving just a little earlier than necessary. This hypothesis could be tested in longitudinal research. This leads to the important and unanswered question whether the FTDr assessment could be performed multiple times without being affected by re-test effects. A repeated assessment of FTDr of patients with AD might be very beneficial considering the progressive course of AD, but also because of additional medical conditions that may affect FTDr only temporarily (e.g. mild stroke, bone fractures).

In order to reduce the number of misclassifications, one could choose not to dichotomize the results into either fit or unfit, but to ‘trichotomize’ the results into three categories, i.e. fit, uncertain or unfit. In trichotomization, two cut-offs are used, meaning that patients scoring below the low cut-off are regarded unfit to drive and patients scoring above the high cut-off are regarded fit to drive, patients with a score in-between the two cut-offs are placed in an uncertain group [70]. It is unclear whether patients in the uncertain group are fit to drive or not, therefore the uncertain group requires further assessment on the road. For the patients in the fit and unfit groups, the fitness-to-drive assessment method may replace the on-road driving assessments in the future.

The on-road assessment was employed as the criterion as it is commonly used and it also represents the current official relicensing procedure for patients with dementia in the Netherlands, however, it has several disadvantages. On-road assessments take place in a changing environment, therefore different tasks will be encountered by different drivers even when driving the same route. In order to improve the comparability of the on-road assessments, a standardized and validated scoring form specifically designed for driving assessment of older drivers was used, called the TRIP [11,54]. Nevertheless, even when using a standardized TRIP form, many circumstances such as adverse weather conditions could impact on on-road assessments. Notably, an on-road assessment is a single, short-term event which makes it vulnerable for coincidental influences [13]. Moreover, patients may receive a doubtful outcome on the on-road assessment if their driving performance is questionable. This outcome is insufficient to renew a driving license, but these patients are invited to a second on-road assessment after taking driving lessons or applying car adaptations. It must be noted that the reliability of the on-road assessment remains unknown what could be a threat to the implications drawn from the present study. In order to investigate the reliability of the on-road assessment, it would be of interest to have participants performing the on-road assessment several times to explore the level of agreement between on-road assessments within subjects. Related to this, a follow-up study would be helpful to determine whether the prediction in the current study corresponds to driving behaviour in real-life situations, e.g. a naturalistic driving study.

A limitation inherent to the used analyses is the problem of capitalisation on chance [71]. Although the initial number of participants (81 patients with AD) is rather high for this type of research [10,12,14,15], the number of participants with usable driving simulator data is smaller. The large number of tests and measures may be problematic as predictors may be identified by chance on the basis of the present sample, resulting in invalid conclusions for the population of drivers with AD. Therefore, a replication of this study on an independent patient sample would be desirable. The current study provides indications which variables differentiate well between fit and unfit drivers with AD, but the method is not ready to be applied until the findings are replicated in an independent patient sample.

For the current study, patients with AD were selected. Nonetheless, patients with other aetiologies of dementia may be impaired in driving as well. The methodology applied in the current study should therefore be employed in studies on FTDr on patients with other aetiologies than AD, e.g. vascular dementia, frontotemporal dementia and Parkinson’s dementia. This is relevant as it was suggested recently that other predictor variables may play a role in the prediction of FTDr in other aetiologies, because symptoms and prognoses of other aetiologies of dementia differ from AD [13].

## Conclusions

Measures from clinical interviews, neuropsychological assessment and driving simulator rides were found to be predictive of on-road driving performance. When comparing the three types of assessments, neuropsychological assessment provided the best prediction of FTDr followed by driving simulator rides and clinical interviews. An even better prediction of FTDr was achieved when combining all three types of assessments. If the results can be replicated in an independent sample of patients with AD, the developed method may be used to advise patients with AD and their family members about FTDr.

## Supporting Information

S1 Appendix Protocol. (DOCX)
