# Supplementary material for: Prediction of Fitness to Drive in Patients with Alzheimer's Dementia
Source: PLoS One. 2016 Feb 24;11(2):e0149566. doi: 10.1371/journal.pone.0149566 (PMC4766198; doi:10.1371/journal.pone.0149566)
Supplement: S1 Appendix — (DOCX) [file pone.0149566.s001.docx]

**Appendix 1 Protocol**

**Inviting patients**

You have received a patient referral from a doctor or the patient has signed up themselves.

Send the patient the information letter along with the participation form and informed consent form by mail, unless the doctor has already given the patient an information letter, participation form, and informed consent form. Wait until the participation form and informed consent form are sent back. If you have not received anything after two weeks call the patient to inquire whether they have forgotten to send the forms. After sign up is completed you can schedule the patient and call them to make an appointment. Afterwards send a confirmation by mail along with a copy of the Driving questionnaire. The patient should fill this in and bring it along on the day of testing. Also mention in the invitation that the patient should bring a list of his medication.

**Preparation for the day of testing**

Two researchers are required to be present on the day of testing:

- The anamnesis supervisor will administer the heteroanamnesis and anamnesis. This supervisor will also be present during the visual field test and driving simulator tests.
- The testing supervisor will administer the neuropsychological tests and is also present during the driving simulator tests.

Both researchers are involved in the driving simulator section. This creates the opportunity for discussion on the driving behaviour and interpretation thereof.

Make sure all of the following items are printed out, write the participant number in the right top corner of each sheet, and place them in the correct order:

- Informed consent form (already filled in by the patient)
- CDR-form
- Addition heteroanamnesis
- Addition anamnesis
- Driving questionnaire (blank version in case the patient did not bring their own)
- Log
- MMSE form, sheet with the sentence to read and illustration, 1 blank A4 sheet
- Trailmaking example page (pre-folded), Trailmaking A and Trailmaking B
- Instruction for visual construction drawings and 2 blank A4 sheets
- Mazes: practice maze, maze 1 and 2, plus table to write down scores (pre-folded)
- NLV
- Motor skills tests form (2 A4 sheets)
- Observation list Researchers’ observations
- Optional: Blank sheet to write down necessary patient details

Write down in the log which details you still need to ask the patient about (e.g. the informed consent form if you have not received this yet). Write down the patient’s contact details on a separate piece of paper to secure it is not scanned in along with the other documents for the database.

Turn on the computers in the designated research area. Both computers must be switched on (if they are not already on).

Make sure to have the following items ready:

- Stopwatch with timer (for administration of Trailmaking A and B, mazes and motor skill tests)
- Pen to make notes with (may also be used for scoring tests)
- Pen the patient may use to draw with (for MMSE, Trailmaking, Visual construction drawings, and maze task)
- Two pens (extra)
- Wristwatch (for MMSE)

**Patient reception**

Welcome the patient and accompanying close relation. Offer them something to drink. Explain that today they will investigate the patient’s driving fitness by using neuropsychological tests and driving simulator tests. ‘We will also ask some general questions regarding memory and driving and we will perform a number of eye tests. In order to paint an accurate picture we always talk to someone who knows you well (the person accompanying you). One researcher will start with that, while the other researcher will complete some tests with you. ‘Ask the accompanying close relation what their plans are. If they decide to leave after the heteroanamnesis inform them of the fact that the patient will be done in approximately 3.5 hours and will be ready to be picked up (if they are unable to go home independently). If they intend to stay close by, ask whether they want to accompany the patient on a break halfway through the test day. Propose to have a break around … o’clock (approximately halfway through the day). You can have coffee/lunch together. We will evaluate how far we have progressed and provide an estimate of how much more time we will need.’ In the meanwhile (until the break) the accompanying relation can engage in other activities (e.g. walk through the city).

‘Is everything clear? Do you have any questions?‘

Subsequently ask the patient if they brought the filled in Driving questionnaire and a list of their medication. Ask whether the questionnaire was filled in together. Also ask if you can make a copy of the list of medication. If there was still information missing (you should have this written down in the log), ask this information from the patient and caretaker.

In case you have not received an informed consent form yet, ask about this and sign the form yourself. Make sure not to mention today’s date. Otherwise, don’t fill it in yet.

If there are no further questions the anamnesis supervisor will accompany the caretaker to a different room for the heteroanamnesis (bring the (filled in) questionnaire, the CDR-worksheet, and heteroanamnesis addition) and the testing supervisor will start with the tests.

**Heteroanamnesis (administered by anamnesis supervisor)**

Tell the caretaker that you will ask them a number of questions on the daily functioning of the patient and the patient’s driving behaviour. First we will start with the daily functioning.

**CDR**

Tell the caretaker that you want to map the patient’s daily functioning according to a standardized questionnaire and that you will therefore ask the questions in a set order. The questions may therefore not come across entirely natural.

Now administer the heteroanamnesis part of the CDR.

**Questions driving safety**

Tell the caretaker that you would like to ask some more questions regarding the patient’s driving behaviour. Go through the list in ‘Addition heteroanamnesis.doc’ with the caretaker.

**Driving questionnaire**

After the CDR’s heteroanamnesis and additional heteroanamnesis you procure the Driving questionnaire and go through the list with the caretaker. Discuss the most problematic points and ask which (if any) of those have become visibly more difficult after the cognitive problems first surfaced. Ask at which points the caretaker and patient may have disagreed and request clarification in cases of ambiguity and distinctive features.

When you have completed the heteroanamnesis thank the caretaker for the cooperation and discuss where the patient will be taken to for the break or the end of the day.

Notify the testing supervisor of the completion of the heteroanamnesis and the place and time the patient will be taken for their break or after completion of all tests.

**Neuropsychological tests (administered by the testing supervisor)**

Observe the patient carefully. Fill in the observation list during the breaks. Note any striking events.

Invite the patient to take place on a fixed seat at a table. Tell the patient that you will first ask them some questions and then complete a number of tasks involving pen and paper. Subsequently there will be a section administered by computer and finally a number of eye tests. Inform the patient that your colleague will ask them some questions regarding their daily functioning and driving a car. Finally the patient will complete number of test drives in the driving simulator.

Tell the patient that testing may cause signs of exhaustion. The patient is therefore free to indicate when they would like to take a break of have something to drink.

Keep the time in mind when administering the NPO in order to be able to take the patient on their break in a timely manner. After the break you will continue where you left off. Write down when the break took place in the log. Pay attention to the patient to see whether they need another small break in between tests.

Use the break to discuss particularities in the patient, the NPO’s progress, when the anamnesis supervisor’s presence is required again and the expected time remaining. At the end of the break one of the two researchers will pick up the patient and discuss where the patient may be taken to after completion of the tests. Indicate an estimated time of completion.

**MMSE**

‘First I will ask you a number of questions.’

Administer the MMSE according to the MMSE protocol. Fill in how the writing went in the Researcher’s observations.

Comments:

- If the patient appears to be well oriented then you can start with asking for the date. It is not necessary to ask for the year, month, and day separately.
- Question 4 requires a choice between a calculation and spelling a word backwards. Choose the spelling option here, since the CDR already includes calculations.

After completing the test write down any comments from the patient or own observations in the log. Use your initials to indicate that you administered the test.

**Trailmaking A and B**

Show the patient the example of **Trail A** (fold the sheet in half so the example of Trail B is covered up). Tell the patient the following: ‘Here you see a piece of paper with a number of small circles each containing a number. You should use this pencil to connect the numbers AS QUICKLY AS POSSIBLE with a line, starting with 1 and continuing until you have connected all the numbers. Do you understand what needs to happen? Then you can practice first.’

If the practice round is successful offer the large sheet to the patient. Only show the sheet to the patient shortly before starting. Tell the patient “You may now begin” while pointing at the beginning, start the stopwatch and write down the time to completion. Set a maximum of 5 minutes. If the patient is not able to complete the test (e.g. the patient quits during the test or makes too many errors) write this down on the sheet.

If the patient makes an error immediate correction is required, if possible, by saying: “No, you made a mistake, you should connect this one to this one.” (Indicate by pointing.) Let the patient continue after this correction, but log the event afterwards. The stopwatch remains on during the correction.

Now, show the patient the example of **Trail B**. ‘On this piece of paper you will see a number of circles, but this time they contain a mixture of numbers and letters. You will have to connect them as quickly as possible in the following manner: 1 - A - 2 - B - 3 - C ... etc. (demonstrate this without using the pencil). Do you understand what needs to happen? Then you can practice first again.’

If the practice round is successful offer the large sheet to the patient (show the sheet to the patient shortly before starting), “You may now begin” (point at the beginning), start the stopwatch and write down the time to completion. If the patient is not able to complete the test (e.g. the patient quits during the test or makes too many errors) then set a maximum time of 6 minutes.

If the patient makes an error immediate correction is required, if possible, by saying: “No, you made a mistake, you should connect this one to this one.” (Indicate by pointing.) Let the patient continue after this correction, but log the event afterwards. The stopwatch remains on during the correction. If the patient wants to give up coach them into continuing the test. After 5 minutes this is no longer necessary. If the patient wants to quit at this point, this is possible. Write down the time and reason for quitting.

After completing the test write down any comments from the patient or own observations in the log. Use your initials to indicate that you administered the test.

**Visual construction drawings**

The testing supervisor puts down a piece of paper in front of the patient and hands them a pen (per item the patient will be provided with a new sheet of paper, if possible use both sides). Then the testing supervisor will say the following:

1) “Please draw a house”. If the patient does not add any details: ‘Could you add some more details?’. If the patient does not add depth: ‘Could you add some more depth?’.

2) “Now would you please draw a star with 5 points”.

3) “Could you draw a cube here”. If the patient does not understand the word “cube”, the term “block” is also acceptable.

4) “Please draw a clock and put the numbers on there, but not the hands.” When the patient has done this: “Now please draw in the hands at 10 past 11.”

After completing the test write down any comments from the patient or own observations in the log. Use your initials to indicate that you administered the test.

**Mazes**

Tell the patient we will administer another test that involves drawing.

We will complete three mazes during this test. We will start off with a practice maze. Say: “Here you see a maze. You enter the maze on the left side (point at entrance). Try to reach the other side of the maze (point at exit). Do this as quickly as possible, but be careful not to follow dead-end paths. If you made a mistake follow the same path you took until you reach the point where you would have liked to go in a different direction. Try to draw as neatly as possible and avoid touching the lines. Do you understand what needs to happen? Please give it a try.” You can take as much time for the practice maze as needed until the patient understands what they need to do. When they complete the practice maze tell the patient again that they should try to avoid the dead-end paths and not touch the lines when drawing. Show the test mazes only just before starting the test.

Maze 1: “Here you can see a different maze. Complete this figure in the same way as you did with the previous maze. You may start here.”

Maze 2: “This is the final maze. Complete this figure in the same way as you did with the previous maze. You may start here.”

Measure the total time the participant needs individually for each maze (thinking time + drawing). With Maze 1 and 2 the patient is given a maximum of 2 minutes to complete each test. Write down on the Researchers’ observations how the patient drew their lines. Also note how powerful their grip was on the pen and how solid the pressure on the paper was with the pen and paper tasks.

Possible situations:

- If the participant is rushing their drawing “You have enough time. It is important not to touch the lines and to prevent entering dead-end paths.
- If the participant is drawing very slowly: “Try to complete the maze as quickly as possible.”
- If they indicate it is too difficult: “It is difficult indeed, but please try to do as best as you can.” If they give up after multiple attempts to encourage the patient to continue you may mark this maze as incorrect/incomplete. If a maze was completed incorrectly, the patient is still asked to complete the next one (“You can try this other maze.”) and otherwise you may terminate the test.
- If they make multiple mistakes: Repeat the instructions that they should try to avoid the dead-end paths and not touch the lines when drawing.
- If the participant asks where they need to go you may point at the exit. Write this event down in the log.

After completing the test write down any comments from the patient or own observations in the log. Use your initials to indicate that you administered the test.

**NLV**

Hand the patient the NLV word list. Instructions: “Here you can see two columns with words. Are the letters readable for you? You will have to read the words out loud. This test is about pronunciation and not about the time needed to read. You will start here (point to left upper corner) and move your way down the list. You will do the same for the right column (also point at this).” Give the patient ‘correct’ or ‘incorrect’ ratings for every word. If you are unsure whether it was pronounced correctly you may use a question mark to indicate this.

Possible situations:

- If the patient is reading too quickly: “This test does not have a time limit, so there is no need to rush.”
- If the patient pronounces a word with an English pronunciation: “Your pronunciation is correct, but you are reading it in English. Could you try doing it Dutch as well?”

After completing the test write down any comments from the patient or own observations in the log. Use your initials to indicate that you administered the test.

**Motor skills tests**

Make sure you are aware of the arm’s position. See this in the video that is referred to in the extended instructions on these tests. Tell the patient that we will perform a number of short tests in order to look at the movements of hands and feet. Demonstrate what the patient should do per body part. Instruction hands: “As quickly as possible open your right hand completely and close it completely so that it makes a fist (demonstrate). Start and stop on my signal.” Set a timer for 5 seconds. Say ‘start’ when you turn it on and ‘stop’ when the timer reaches zero. Score the number of completed movements in 5 seconds and indicate to which category the results belong. Repeat this process for the left hand. Instruction feet: “Put both of your feet on the ground. Tap the floor with your right foot while leaving your heel on the ground (demonstrate). Do this as quickly as possible. Start and stop at my signal.” Set a timer for 5 seconds. Say ‘start’ when you turn it on and ‘stop’ when the timer reaches zero. Score the number of completed movements in 5 seconds and indicate to which category the results belong. Repeat this process for the left foot. Score the observation category that is applicable to the results in general.

**Proprioception test**

The Proprioception test in printed on the same form as the motor skills tests. Say: ‘Put your hand on the table. Close your eyes.’ Check whether the patient has really closed their eyes. Then say: ‘Touch the tip of your nose with your right index finger.’ Record the time. Note how long it took and choose the appropriate category for the finger placement and movement. Repeat this procedure for the left index finger.

After completing the test write down any comments from the patient or own observations in the log. Use your initials to indicate that you administered the test.

**ATAVT (S1)**

To start this test, double click on the shortcut [Vienna Test System]. First create a new participant by opening the tab ‘List of test persons’ and clicking the button [new]. Enter the participant’s number in the name field. Do not enter a first name, leave this field blank (or add an ‘x’ if it requires input). Ask for the participant’s date of birth. Enter this into the system. Click [OK]. Enter the sex, level of education and language. Click [OK]. Now click on [Tests] and select [ATAVT]. Select version S1, right-hand traffic countries. Click [OK]. Now click [Start test]. If a pop-up appears, click [OK]. Ask the participant if they ever use a computer at home and if necessary reassure them that is not as difficult as it may seem. Remove the standard keyboard from the computer and replace it with the Vienna Test System keyboard. Have the participant take place in front of the computer.

Tell the participant the following: “You may now take place right in front of the computer. Are you sitting alright? You will be briefly shown pictures of various traffic situations throughout the next ten minutes. After every picture the computer will ask you what you have seen. In order to answer these questions you will only have to use these keys (point at keys) of the special keyboard. First you will receive instructions from the computer. If you have questions about this feel free to ask. After these instructions you will be able to practice a few times. The computer will indicate when the real test starts.”

Have the participant go through the instructions, read the instructions out loud if necessary and let them go through the instructions by using the keyboard so that they may get acquainted with the special keyboard. Explain that if the participant wants to correct an answer they may do so by pressing the same key another time to make the previous answer disappear. Demonstrate this during the second practice question if it does not occur naturally. When the participant has gone through the instruction and has no further questions they may start the actual test.

Possible situations:

- If the participant does not remember what they saw, ask them if they really cannot recall anything. If not, have them leave the answer blank. If no answer is selected a yellow window appears that asks if the participant is sure. Have the participant press the green button once more.
- If the participant says that there was nothing on the picture ask them if they are sure, since there is always something on the pictures. If they are sure, have them leave the answer blank. The yellow screen will pop up once more. Have the participant press the green button again.
- If the participant complains that they do not have enough time and that in a real life traffic situations you have more time to assess the situation tell them that it is important to gain insight in the traffic situation really quickly. As a driver you do see the situation for a longer period of time, but very often they will have to make decisions really quickly. This is the reason why the pictures are only visible briefly.
- If the participant wrongly selects all categories because they could hypothetically have been there, tell them that they should only select the categories they actually saw.

After completion of the test choose [print test results] in the pop-up screen. Minimize the Vienna Test System program. Write down any comments from the patient or own observations in the log. Use your initials to indicate that you administered the test.

**Traffic theory test**

Double-click the [TotalCMD] shortcut. Open the driving theory test: c:\ Theorie1\Theorie1_STD_12sec by double clicking. Enter the participant’s number in the name field and press [enter]. For the question ‘12 seconds time limit (Y/N)?’ answer ‘y’. Finally it shows how the output file will be displayed. If it is correct, press ‘y’. The preview picture appears on the screen immediately. If the participant is used to working with a computer they will probably be able to enter the answers themselves. If uncertain, tell the patient that you will help them out. Remove the Vienna Test System keyboard and replace it with the standard computer keyboard.

Have the participant take place straight in front of the computer. Ask whether they can see the picture on the screen clearly and if they can reach the keyboard comfortably. When the participant is seated correctly you can start explaining the task: “Over here you can see a picture of a traffic situation from the point of view of a car driver. This is your steering wheel (point at it). You are looking at the situation through the car’s windshield. You will have to read the question and indicate which answer is correct. There will always be two possibilities. The two options are indicated on the screen here (point at it). Each question is different and so are the accompanying answers. If you want to select answer [1] on a question you must press the [1] key and if you want to select answer [2] press the [2] key (point at the 1 and 2 keys on the numpad). You will have 12 seconds to answer each question. The time is indicated on the bottom of the screen with 12 crosses that disappear with time. If all crosses are gone than your time is up. The picture will then disappear and you will no longer be able to answer. Therefore it is important that you select your answer within 12 seconds. Are you able to do this yourself or would you rather have me do the typing?”

Discuss the practice picture with the patient. Ask them what they would answer. The right answer is ‘No’. Discuss that this is because traffic that is going straight has priority over turning traffic on the same road. Also direct the patient’s attention to the signs on the side of the road, although these do not matter for the answer. ‘The next questions may be more pictures from the car’s perspective, but they can also be about the meaning of traffic signs.’

In case of doubt whether the patient is able to do the typing, have them try it out during the practice question. If necessary you can offer to take over the typing after the practice question. If the person prefers you to do the typing: “Please use the following words when you give your answer: 1 or 2.” Just before you press the space bar alert the patient that the next picture is coming.

For everybody: “You do not have to explain to me what you are seeing and why you made a certain choice, you are only required to answer within the time limit. When you give the answer or when the 12 seconds have passed the picture will disappear from the screen. You may then press the space bar to go to the next question.”

Then press the [space bar]. A screen with the following notice will appear on screen ‘The first pictures are for practice. Space bar starts the test.’ If necessary explain that by pressing the space bar the practice pictures will show up on the screen.

Possible situations during practice:

- The patient has difficulties finding the keys: offer to take over the typing.
- The patient mentions details about what the answer could be and what they are seeing: tell them that this is not necessary and that they only have to indicate whether they choose answer 1 or 2.
- If you think that the patient has not fully understood the task repeat the instructions.

After the practice pictures: “Do you have any questions at this point? If you press the space bar the test will start and the first question will appear on the screen.”

Watch the patient while they complete the test. If they are almost too late (2 crosses remaining) make them aware that they should answer now. You are not allowed to comment on the correctness of the answer, but you are allowed to help the patient answer in time and complement them on their pace. If finding the right keys takes too much time you should offer the patient to press the keys for them.

Press the space bar to close the program.

After completion of the test ask how the participant thought it went. Write down any observations you made during the test in the log, especially whether you did the typing or not. Use your initials to indicate that you administered the test.

**Hazard perception test**

Double-click the [TotalCMD] shortcut. Open the hazard perception test: c:\ gevaar1\Gevaar1_STD by double clicking. Enter the participant’s number in the name field and press [enter]. For the question ‘8 seconds time limit (Y/N)?’ answer ‘y’. Finally it shows how the output file will be displayed. If it is correct, press ‘y’. The preview picture appears on the screen immediately. If the participant is used to working with a computer they will probably be able to enter the answers themselves. If uncertain, tell the patient that you will help them out.

Have the participant take place straight in front of the computer. Ask whether they can see the picture on the screen clearly and if they can reach the keyboard comfortably. When the participant is seated correctly you can start explaining the task: “Here you can see a traffic situation as seen from a car driver’s point of view. You can see the car’s dashboard (point at screen), with a speedometer (point at it) that indicates the car’s speed and (if necessary) turn signals [point out where this will be visible]. You also see the inside mirror (point at it) so you are able to see what happens behind the car. You can also see what is happening through the windshield. You have to indicate what you would do if you were the driver of this car. You have three options: brake, this means a rapid speed decline, release the gas pedal, this means a slow speed decline, or do nothing, which means maintaining the same speed. These three options are indicated on the screen here (point at it). For braking press [1], for releasing the gas pedal press [2], and for changing nothing press [3] (point at the keys on the numpad). You will have 8 seconds to answer each question. The time is indicated on the bottom of the screen with 8 crosses that disappear with time. If all crosses are gone than your time is up. The picture will then disappear and you will no longer be able to answer. Therefore it is important that you select your answer within 8 seconds. Are you able to do this yourself or would you rather have me do the typing?”

Discuss the practice picture with the patient. Ask them what they would do in this situation. The answer should be releasing the gas pedal or braking (both answers are correct), because your view of the right is obstructed.

In case of doubt whether the patient is able to do the typing, have them try it out during the practice question. If necessary you can offer to take over the typing after the practice question. If the person prefers you to do the typing: “Please use the following words when you give your answer: brake, release gas, or do nothing.” Just before you press the space bar alert the patient that the next picture is coming.

For everybody: “You do not have to explain to what you are seeing and why you made a certain choice, you are only required to answer within the time limit. When you give the answer or when the 8 seconds have passed the picture will disappear from the screen. You may then press the space bar to go to the next question.”

Then press the [space bar]. A screen with the following notice will appear on screen ‘The first pictures are for practice. Space bar starts the test.’ If necessary explain that by pressing the space bar the practice pictures will show up on the screen.

Possible situations during practice:

- The patient has difficulties finding the keys: offer to take over the typing.
- The patient mentions details about what the answer could be and what they are seeing: tell them that this is not necessary and that they only have to indicate whether they would brake, release gas, or do nothing in that situation.
- If you think that the patient has not fully understood the task repeat the instructions.

After the practice pictures: “Do you have any questions at this point? If you press the space bar the test will start and the first question will appear on the screen.”

Watch the patient while they complete the test. If they are almost too late (2 crosses remaining) make them aware that they should answer now. You are not allowed to comment on the correctness of the answer, but you are allowed to help the patient answer in time and complement them on their pace. If finding the right keys takes too much time you should offer the patient to press the keys for them.

Press the space bar to close the program.

After completion of the test ask how the participant thought it went. Write down any observations you made during the test in the log, especially whether you did the typing or not. Use your initials to indicate that you administered the test.

**RT (S1, S2, and S3)**

**S1: Simple reaction time with yellow dot.** Open the Vienna Test System again. Navigate to the [testing] tab. Click on [Previous test person]. Select [RT]. Select [S1]. Click [OK]. Click [Start test]. If a pop-up appears, click [OK]. Place the Vienna Test System keyboard in front of the participant again. Tell the participant: “You can now read the instructions on the screen. It is important that you operate the buttons with the same finger. You are only allowed to use one hand. Which hand has your preference? Then you should use this hand. After reading the instructions there will be a number of examples. After this the real test will start.”

If necessary the testing supervisor may read the instructions from the screen to the participant. After the practice run ask whether the participant has understood the task and if they are ready for the real test. You are also able to estimate this based on the participant’s achievements during their practice. If the participant did not fully understand the instructions press [Esc] [F5] on the standard keyboard, which brings you to a menu in which you can select an option to show the instructions again. After a third practice run you will have the participant start with the real test, even if the participant’s level is still low. If at all possible try to administer the test.

Possible situations:

- If the participant asks if they have to press now (when a yellow dot is visible) you may help them during the first stimulus, but not after this.
- If the participant presses the wrong button (often they accidentally press the grey button) correct the participant.
- If the participant asks for confirmation during the test or if you notice that they are not very confident in their results, you are allowed to tell them that they are doing well after a good reaction in order to make them feel at ease.
- If the participant starts talking ask them if they can fully focus on the task.

Try to sit next to the participant as quietly as possible. After completion choose to print the results.

**S2: Simple reaction time to sound.** The process of administering this test is exactly the same as S1. Therefore the instructions are also the same. During the instructions ask whether the patient is able to hear the sound properly.

**S3: Choice reaction time with yellow dot and sound.** Again, the process of administering this test is exactly the same. Tell the participant that this test may be more difficult. During practice observe the participant to make sure they completely understood the instructions.

After completion of the three tests write down your initials to indicate that you administered the test in the log and note your observations in the appropriate section. If the participant wanted to practice multiple times, needed affirmation, pressed the wrong button or forgot the assignment during the test in S3, you should write this down. Also note in the Researcher’s observations how the pressure on the golden button was and whether the patient has to look at their fingers in order to press the correct buttons.

**DT (S1)**

Navigate to the [testing] tab and click [Previous test person]. Click [DT]. Then click ‘version S1’, short version with adaptive stimulus presentation (all stimulus modi). Click [OK]. Click [Start test]. If a pop-up appears, click [OK]. Ask the participant if they are still sitting in a good position to the keyboard. Inform them that they may use two hands in this task. Ask the participant if they are able to reach the pedals with their feet and adjust their position if necessary. Then have the participant go through the instructions. Read the instructions out loud if the participant is more comfortable with this. Where necessary, clarify the text and help the participant during practice by telling them what to do when a stimulus appears on the screen. In the case of sounds it might be useful to indicate that the top button should be pressed for a high tone and the bottom button for a low tone. This could make it more logical for the participant.

Possible situation during practice:

- If the participant is unable to complete the practice independently after the first practice round, press [Esc] [F5] in order to restart the practice.
- If the participant makes to many errors a red pop-up will appear on screen. Press [Esc] [F5] to restart the practice.

After pressing [Esc] [F5] a pop-up appears on the screen. Press [F1] in this screen (restart the instructions). In the next screen you can choose from which point to start the instruction again. You can also choose to just redo the practice exercise. You will have to determine yourself which is most beneficial to the participant.

You can let the participant practice three times until continuing to the real test. Before starting the test tell the participant that the test takes a bit longer, 4 minutes. Also remind the participant to remain calm during the test and not to feel hurried.

Try to speak as little as possible during the test.

Possible situations:

- If the participant suddenly stops, attempt to persuade them to move again. Tell them that if they react slower the test will adapt to this and slow down as well, so they should try to take it slow.
- If the participant asks for affirmation you may provide this for the first stimulus only. Afterwards you should not do this.

You are allowed to praise the participant if they react calmly. Do not tell them which button to press, but do praise their pace and posture.

After completing the test choose to print the results. Write down your initials for the test and comments on the participant’s performance, at least whether they had to practice more than once. Possible observations to include: no longer reacted with the pedals, was startled by the sounds (thought it was an error), etc.

Minimize the Vienna Test System program.

**Eye tests**

**ETDRS chart for visual acuity.** Turn all lamps on to create a sufficiently illuminated room. Have the participant stand 3 meters from the chart. They are allowed to look with both eyes. If the participant is not wearing glasses ask if they ever wear glasses and when. If the participant is nearsighted ask them to put on their glasses. Start with the row with letters at 10M. Ask the participant to read out the letters in this row. If they are unable to read these move up a row until you reach a row the participant is able to read out fully. If the participant is able to read the letter at 10M, then move down a row at a time until they make a mistake. Choose the left rows at the split. If you notice one error in the left row you can then try the right row at the same height. Determine the row with the smallest letters that did not contain errors. Divide the number 3 with the number indicated on the left of these rows (the M number). This is the value for visual acuity. Write this down in the log. Write down your initials and add comments if applicable.

**Hamilton-Veale’s chart for contrast sensitivity.** Have the participant stand 1 meter from the chart. Tell them that there are letters on this chart as well. Start at number 9 and ask the participant to read out the letters. If they are unable to read this, go down a number until the patient reads two letters next to a number without errors. If the participant is able to read the letters at number 9 then move up a number until the participant is unable to read. Ask them to read the next one anyway and return to the previous number to make sure they still cannot read it. Write the highest errorless number in the log and add /16 together with your initials.

**Visual field measurement.** Ask the anamnesis supervisor to join you in the test room. The patient keeps the protractor placed on his nose and looks at the protrusion at 0ᵒ. The anamnesis supervisor checks whether the patient keeps looking at this point. Show the patient the piece of wood with the dot they have to pay attention to. The test supervisor stands behind the patient and moves the piece of wood with the dot from 90ᵒ along the bow. The patient will speak up when the dot appears in the field of vision. Measure how many degrees this is and repeat this twice from both sides. Write down how many degrees you measured (angle α) for right and left, your initials, and any comments in the log.

Turn on the screens for the driving simulator. These need at least 10 minutes to start up properly. Remove the Vienna Test System keyboard and replace it with the regular keyboard again. Click on the [Start simulator] shortcut in order to boot the software needed to control the driving simulator.

**Anamnesis (administered by the anamnesis supervisor)**

Have the patient take place at the table. Explain to the patient that you would like to ask them some questions about their home situation and car driving.

**CDR anamnesis**

Start with the anamnesis part of the CDR according to the order of the CDR.

**Driving behaviour**

Handle the additional heteroanamnesis. Discuss particularly striking parts of the driving behavior that came forward in the FTDS questionnaire with the patient’s caretaker.

**Driving simulator tests (administered by both researchers)**

During this part both researchers will be present. Discuss the division of tasks beforehand. Usually the anamnesis supervisor will be the one operating the computer while the test supervisor explains the test drives to the patient.

Make sure that the two lamps behind the screens are on and the big light in the room off. Check whether both computers and all screens are on.

Have the patient take place in the driving simulator chair. Make sure the patient is sitting comfortably, has their seatbelt on, and can reach the pedals with their feet. Readjust the chair if necessary. Have the patient press down on a pedal. This is important, because it ensures the pedals are properly set and calibrated. Have the patient practice using the gas and brakes so they will not have to do this later on. Say: “Press down the gas pedal. Press down the brakes. Press down the gas once more. Press down the brakes once more. Gas. Brakes.” If necessary explain which pedal controls the gas and which one the brakes. Continue until the patient has pressed both pedals properly 3 times. Note down how the practicing went and how often pressing the pedals was necessary in the Researcher’s observations.

Take the motor skills tests sheet and stopwatch to administer the neck flexibility test.

Tell the patient: “First we will perform a short test to determine whether you are able to look over your shoulder properly. Keep your hands on the steering wheel and look straight ahead. I will now stand behind you.” Stand 3 meters behind the patient and put up a random number of fingers straight under your chin. Instruction: “Do not let go of the steering wheel. Look over your right shoulder as if you were parallel parking. Tell me how many fingers I am holding up.” Start the stopwatch. Stop the counter when the patient answers and write down this time. Also note whether the answer was correct or whether the patient was unable to see it. Repeat this for the left shoulder.

Walk back to the patient and explain what testing in the driving simulator will be like: “Driving in the driving simulator feels different from driving in a normal car, because (the feeling of) motion is missing. This may feel a bit odd, but that happened to most people and is not bad. People may experience motion sickness while driving in the driving simulator. Please, tell us if you feel dizzy or nauseous, then we will stop the ride. We will go on a short walk with you and drink a glass of water in order to make you feel better. If you start feeling nauseous this will not influence the advice we will give you. The only consequence is that we will not be able to complete the driving simulator tests drives. You have already completed the other parts of the research. Shall we begin?” Observe the patient carefully during the test drives and fill in the Researcher’s observations.

**Swingdrive with automatic increase in speed**

**Computer details.** Enter the patient’s FitCI number in the ‘Subject Ident’ field and end with a [tab]. Click on [start simulation]. Another window will appear on the screen. Now you have to select the correct course: c\: Stsoftware\Simulator\Scripts\FitCI\Swingdrive\Swingdrive_FitCI.scn. The button [pause] is activated. Click this button to deactivate it. On the three screens you will now see video from the test drive. Now you explain to the patient what will happen throughout this course. Select in the window “scenarios”[0001 START Fixed speeds]. Then click [Start scenario]. The car will start driving automatically. You do not need to use the key or gears.

**Patient communication.** “We start with a relatively simple ride to get used to driving in a driving simulator. You will drive on this rural road. In this ride, the only thing you have to do is steering, using the accelerator and/or the brake pedal is not allowed. There will be oncoming traffic on the opposite lane, but they will keep driving on the opposite lane and you will not experience any problems with these cars. You are the only one driving in your lane, no cars will be driving behind you or in front of you. You will only have to keep the car in your own lane. You can look in your mirrors (point at all three). Can you see the speedometer on your screen? Above the speedometer you can also see your speed displayed in numbers. The computer controls the speed at which you drive. You start driving at 50km/u and from there on the computer will speed up the pace with 10 km/u over time until you drive at 100 km/u. At the end of the ride a computer voice will ask you to park the car. Do you have any questions so far? We will start the ride now.”

**During the drive.** Try to make the patient feel comfortable. If the patient indicates that it feels odd assure them that this is a normal reaction. This is why this test is done first: so that the patient can get used to driving in a driving simulator.

**After the drive.** Tell the patient to park the car on the side of the road and slowly decelerate. Click [stop] in the window ‘scenarios’. Click [yes] in a different window if asked for confirmation. Ask the patient if he or she experienced any difficulties while driving (or ask the question ‘how did it go?’). If they indicate that steering feels very different confirm this. Tell them that we will take this into account, but that they should try their best. Ask how the patient feels. If the patient feels nauseous or very dizzy you will have to stop. If not, then you may continue to the next test drive. Write comments of the patient and your own observations down in the log. Write down if you needed to repeat any instructions. Take these steps after every ride.

**Swingdrive with free speed choice**

**Computer details.** Make sure the gear stick is in neutral and the car key is turned left. Click on [start simulation]. Another window will appear on the screen. Now you have to select the correct course: c\: Stsoftware\Simulator2\Scripts\FitCI\Swingdrive\Swingdrive_FitCI.scn. The button [pause] is activated. Click this button to deactivate it. On the three screens you will now see video from the test drive. Now you explain to the patient what will happen throughout this course. Select in the window “scenarios”[0002 START Free speed choice]. Then click [Start scenario]. The patient can start the car by turning the key like they would in a real car and by pushing the gear unto 2 or 4. During the drive the patient does not need to touch this anymore.

**Patient communication.** “This ride is almost the same as the previous ride, but there is one important difference: This time the computer does not control your speed while driving. You have to control the speed using the accelerator. If you wish to drive at a slower speed just let the accelerator go (a little bit). You have to choose a speed comparable to the speed you would drive when driving in your own car on a similar road. At a halfway point in the course a computer voice will say: ‘Now you’re in a hurry. Speed up the pace, but still drive at a safe and responsible speed.’ When you hear this, you have to drive at a higher speed than before like you’re in a hurry, like you’re too late for an appointment. When choosing this speed, keep in mind that you need to maintain track of the lane without swerving too much. So this ride is about driving at two different speeds: driving at a ‘normal’ speed and driving at a faster speed. You will start the car yourself (turn the key or push the button). It has an automatic gearbox, if you put the gear stick in the second position it is in ‘drive’. You do not have to touch the gear stick anymore during the ride. Do you have any questions so far? We will start the ride now.”

**During the drive.** Make sure the patient feels comfortable.

**After the drive.** After the drive tell the patient to park the car on the side of the road and slowly decelerate. Click [stop] in the window ‘scenarios’. Click [yes] in a different window if asked for confirmation. Tell the patient to put the car in neutral and turn the key back to its original position. Write comments of the patient and your own observations down in the log. Write down if you needed to repeat any instructions. Take these steps after every ride.

**Intersections 1**

**Computer details.** Add ‘a’ to the ‘Subject Ident’ and end with a [tab]. Click on [start simulation]. Another window will appear on the screen. Now you have to select the correct course: c\: Stsoftware\Simulator2\Scripts\FitCI\Intersections\ Intersections_FitCI.scn. The button [pause] is activated. Click this button to deactivate it. On the three screens you will now see video from the test drive. Now you explain to the patient what will happen throughout this course. Select in the window “scenarios” [0001 START Drive]. Then click [Start scenario]. Tell the patient they can start the car and put the gear stick into 2 or 4.

**Patient communication.** “The next ride is a different ride in a rural environment. The most important difference between this ride and the previous ride is that now you will encounter multiple intersections. You always have to drive straight ahead, never take a turn. The right of way on these crossings is either regulated by traffic signs or lights located near the intersections or they are uncontrolled intersections. It will take a little longer for you to notice the intersections and signs than would be the case in real life. Therefore it is advisable to encounter these intersections and traffic signs at a slower speed to get used to this, since you have to recognize the traffic signs first and subsequently have to deal with traffic. You also have to obey speed limit signs. You may drive slower than the maximum speed, but you may never drive faster than the speed limit. You will have to use the accelerator and the brake. Do you have any questions so far? We will start the ride now.”

**During the drive.** During the drive you may answer the patient’s questions. If they show signs of uncertainty try to reassure them. Since this is the first ride you are allowed to help the patient a little. Observe the patient’s looking behavior and write any observations down in the log. Also note down strange maneuvers a patient may make.

**After the drive.** After the drive tell the patient to park the car on the side of the road and slowly decelerate. Click [stop] in the window ‘scenarios’. Click [yes] in a different window if asked for confirmation. Tell the patient to put the car in neutral and turn the key back to its original position. Ask the patient how they thought it went. Also ask them how they feel. Write down observations you made during the test drive in the log. Also add the extent to which you had to help the patient.

**Intersections 2**

**Computer details.** Replace ‘a’ by ‘b’ in the ‘Subject Ident’ and end with a [tab]. Click on [start simulation]. Another window will appear on the screen. Now you have to select the correct course: c\: Stsoftware\Simulator2\Scripts\FitCI\Intersections\ Intersections_FitCI.scn. The button [pause] is activated. Click this button to deactivate it. On the three screens you will now see video from the test drive. Now you explain to the patient what will happen throughout this course. Select in the window “scenarios” [0001 START Drive]. Then click [Start scenario]. Tell the patient they can start the car and put the gear stick into 2 or 4.

**Patient communication.** “This ride is exactly the same as the previous ride, so you know what to expect. Pay close attention to the signs on the side of the road and drive like you would in your own car.” If you helped the patient in Intersections 1 tell them that you will no longer do this and that the patient will have to complete the test entirely independently. “Do you have any questions so far? We will start the ride now.”

**During the drive.** During the second intersections drive you are not allowed to help the patient. They will have to complete it entirely independently. Observe the patient’s looking behavior and write any observations with regards to this or unusual maneuvers down in the log.

**After the drive.** After the drive tell the patient to park the car on the side of the road and slowly decelerate. Click [stop] in the window ‘scenarios’. Click [yes] in a different window if asked for confirmation. Tell the patient to put the car in neutral and turn the key back to its original position. Ask the patient how they thought it went. Also ask them how they feel. Write down observations you made during the test drive in the log.

**Merging**

**Computer details.** Change the ‘Subject Ident’ back to just the FitCI number and end with a [tab]. Click on [start simulation]. Another window will appear on the screen. Now you have to select the correct course: c\: Stsoftware\Simulator2\Scripts\FitCI\Merging\ Merging_FitCI.scn. The button [pause] is activated. Click this button to deactivate it. On the three screens you will now see video from the test drive. Now you explain to the patient what will happen throughout this course. Select in the window “scenarios” [0001 START task]. Then click [Start scenario]. Tell the patient they can start the car and put the gear stick into 2 or 4.

**Patient communication.** “We will now start with the final test drive. The purpose of this ride is to merge into the motorway traffic. You are located on the ramp and want to merge unto the highway. Right now you are standing in the left hand lane (point at lane), as the right hand lane is the emergency lane (point at lane). The highway is here (point at highway) behind this row of trees. Adjust your speed to the other traffic. The first assignment is to merge into the moving traffic on the highway. Then you are instructed by the computer voice to pass one car in front of you and subsequently steer back to the right hand lane. The final assignment is to exit the highway. The test drive ends on this exit. It is important that you drive as you would do in your own car on the highway, keeping other traffic in mind. Do you have any questions so far? We will start the ride now. You are located on the ramp to the highway. You will have to speed up right away to reach an appropriate speed for merging.”

**During the drive.** If necessary encourage the patient to drive faster. The computer indicates when the patient has to overtake. If the patient does not follow these directions tell them the assignment again. Do not indicate when the patient should merge with the other lane. If the patient has other questions you are allowed to answer those.

If the patient is driving extremely slowly, encourage them to drive faster. If after this they still do not increase their speed do not mention this again.

**After the drive.** After the drive tell the patient to park the car on the side of the road and slowly decelerate. Click [stop] in the window ‘scenarios’. Click [yes] in a different window if asked for confirmation. Tell the patient to put the car in neutral and turn the key back to its original position. Ask the patient how they thought it went. Also ask them how they feel. Write down observations you made during the test drive in the log.

Close the program.
